# JunB is required for CD8^+^ T cell responses to acute infections

**DOI:** 10.1093/intimm/dxae063

**Published:** 2024-10-19

**Authors:** Shukla Sarkar, Naoyuki Taira, Tsung-Han Hsieh, Hsiao-Chiao Chien, Masato Hirota, Shin-ichi Koizumi, Daiki Sasaki, Miho Tamai, Yu Seto, Mio Miyagi, Hiroki Ishikawa

**Affiliations:** Immune Signal Unit, Okinawa Institute of Science and Technology, Graduate University (OIST), Onna-son, Okinawa 904-0495, Japan; Immune Signal Unit, Okinawa Institute of Science and Technology, Graduate University (OIST), Onna-son, Okinawa 904-0495, Japan; Immune Signal Unit, Okinawa Institute of Science and Technology, Graduate University (OIST), Onna-son, Okinawa 904-0495, Japan; Immune Signal Unit, Okinawa Institute of Science and Technology, Graduate University (OIST), Onna-son, Okinawa 904-0495, Japan; Immune Signal Unit, Okinawa Institute of Science and Technology, Graduate University (OIST), Onna-son, Okinawa 904-0495, Japan; Immune Signal Unit, Okinawa Institute of Science and Technology, Graduate University (OIST), Onna-son, Okinawa 904-0495, Japan; Immune Signal Unit, Okinawa Institute of Science and Technology, Graduate University (OIST), Onna-son, Okinawa 904-0495, Japan; Immune Signal Unit, Okinawa Institute of Science and Technology, Graduate University (OIST), Onna-son, Okinawa 904-0495, Japan; Immune Signal Unit, Okinawa Institute of Science and Technology, Graduate University (OIST), Onna-son, Okinawa 904-0495, Japan; Immune Signal Unit, Okinawa Institute of Science and Technology, Graduate University (OIST), Onna-son, Okinawa 904-0495, Japan; Immune Signal Unit, Okinawa Institute of Science and Technology, Graduate University (OIST), Onna-son, Okinawa 904-0495, Japan

**Keywords:** AP-1, apoptosis, co-inhibitory molecules, effector and memory CD8^+^ T cells, glycolysis

## Abstract

Basic-leucine zipper transcription factor ATF-like (BATF) and interferon regulatory factor 4 (IRF4) are crucial transcription factors for the generation of cytotoxic effector and memory CD8^+^ T cells. JunB is required for expression of genes controlled by BATF and IRF4 in CD4^+^ T cell responses, but the role of JunB in CD8^+^ T cells remains unknown. Here, we demonstrate that JunB is essential for cytotoxic CD8^+^ T cell responses. JunB expression is transiently induced, depending on the T cell receptor signal strength. JunB deficiency severely impairs the clonal expansion of effector CD8^+^ T cells in response to acute infection with *Listeria monocytogenes*. *Junb*-deficient CD8^+^ T cells fail to control transcription and chromatin accessibility of a specific set of genes regulated by BATF and IRF4, resulting in impaired cell survival, glycolysis, and cytotoxic CD8^+^ T cell differentiation. Furthermore, JunB deficiency enhances the expression of co-inhibitory receptors, including programmed cell death 1 (PD-1) and T cell immunoglobulin mucin-3 (TIM3) upon activation of naive CD8^+^ T cells. These results indicate that JunB, in collaboration with BATF and IRF4, promotes multiple key events in the early stage of cytotoxic CD8^+^ T cell responses.

## Teaser

JunB controls the expansion of cytotoxic CD8+ T cell in acute infection

## Graphical Abstract

**Graphical Abstract:**
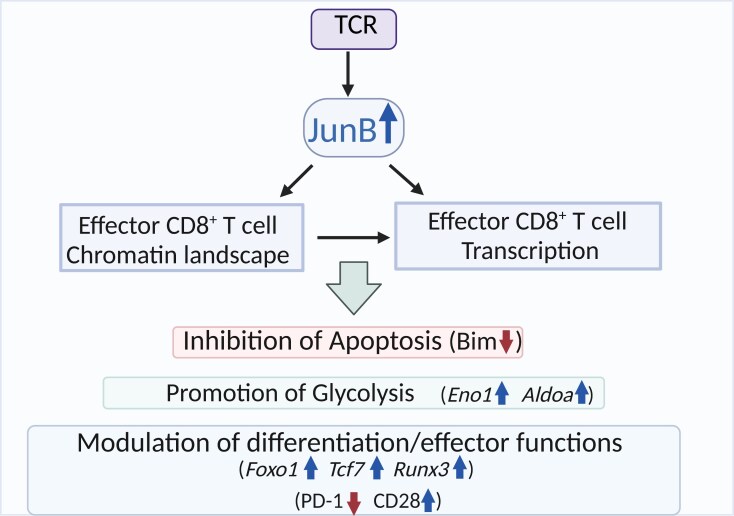

## Introduction

Upon acute infection with intracellular pathogens or vaccination, naive CD8^+^ T cells are activated by signals from T cell receptors (TCRs), co-stimulatory molecules, and inflammatory cytokines, inducing them to undergo massive cell proliferation. This process is promoted by metabolic reprogramming from catabolic mitochondrial oxidative phosphorylation to aerobic glycolysis to support the rapid growth of effector CD8^+^ T cells (1). This clonal expansion results in the generation of abundant cytotoxic effector CD8^+^ T cells, which kill infected cells (2). After pathogen clearance, the majority of effector CD8^+^ T cells die, while the remaining ~5%–10% persist as memory CD8^+^ T cells. During the early clonal expansion phase, memory precursor effector cells (MPECs), which exhibit higher potential to differentiate into memory CD8^+^ T cells, and short-lived effector cells (SLECs), which have higher proliferative ability, are generated (3). These two populations differ in expression of killer cell lectin-like receptor G1 (KLRG1) and IL-7 receptor alpha (CD127) (KLRG1^lo^CD127^hi^ MPECs vs KLRG1^hi^CD127^lo^ SLECs) (4–6). Differentiation of effector and memory CD8^+^ T cells is controlled by multiple transcription factors (TFs). For example, SLEC differentiation is facilitated by the T-box TF (T-BET), B lymphocyte-induced maturation protein 1 (BLIMP1), inhibitor of DNA binding 2 (ID2), and zinc finger E-Box binding homeobox 2 (ZEB2), while it is inhibited by nuclear receptor subfamily 4A (NR4A1) (5, 7–13). In contrast, memory CD8^+^ T cell differentiation is supported by eomesodermin (EOMES), B cell lymphoma protein 6 (BCL6), ID3, ZEB1, signal transducer and activator of transcription 3 (STAT3), T cell factor 1 (TCF1), and forkhead box O 1 (FOXO1), while it is suppressed by NR4A3 (11, 14–19).

Basic-leucine zipper TF ATF-like (BATF) and interferon regulatory factor 4 (IRF4), induced by TCR signaling, promote responses of CD8^+^ T cells (20, 21) as well as CD4^+^ T helper (22–25) and regulatory T (Treg) cells (26). BATF heterodimerizes with Jun family members, and in turn, forms a trimeric TF complex with IRF4. This trimeric complex binds to AP-1-IRF composite element (AICE) motifs (27, 28). In CD8^+^ T cell responses to acute infection, BATF and IRF4 support clonal expansion of TCR-stimulated CD8^+^ T cells by promoting cell proliferation, survival, and metabolic reprogramming (20, 29, 30). Additionally, they facilitate the differentiation of effector CD8^+^ T cells by controlling the expression of key genes in this process, including *Tbx21* (encoding T-BET) and *PR/SET domain 1* (*Prdm1* encoding BLIMP1) (29). Moreover, BATF contributes to the regulation of chromatin remodeling in cytotoxic CD8^+^ T cell responses *in vivo* (31), as well as Th17 differentiation *in vitro* (32).

JunB collaborates with BATF and IRF4 to facilitate pathogenic Th17 differentiation (33–35) and to support the survival of Th1, Th2, and eTreg cells (36, 37). JunB is necessary for the regulation of a subset of target genes for BATF and IRF4, such as Th17 signature genes and pro-apoptotic *Bcl2l11* (encoding Bim), in Th subsets and eTreg cells (33, 34, 36, 37). Given such functional interaction of JunB, BATF, and IRF4 in CD4^+^ T cells, we speculated that JunB may also contribute to transcriptional regulation of CD8^+^ T cell responses. In this study, we demonstrate that JunB is an essential TF for cytotoxic CD8^+^ T cell responses.

## Methods

### Mice

*Junb* ^fl/fl^ mice were generated previously (33). CD4-Cre (stock #017336), B6SJL (stock# 002014), and OT-I TCR transgenic mice (stock #003831) were purchased from the Jackson Laboratory. All mice were maintained on a C57BL/6 background under specific pathogen-free conditions. Sex-matched mice, aged 6–18 weeks, were used for experiments. The Animal Care and Use Committee of the Okinawa Institute of Science and Technology Graduate School approved all animal protocols for this study.

### Antibodies

For fluorescence-activated cell sorter (FACS) and flow cytometry analysis, the following antibodies were used: anti-CD8 (53-6.7, Biolegend, 1:400 dilution), anti-CD3 (17A2, Biolegend, 1:400), anti-CD44 (IM7, Biolegend, 1:400), anti-CD62L (MEL-14, Biolegend, 1:400), anti-CD45.1 (A20, Biolegend, 1:400), anti-CD45.2 (104, Biolegend, 1:400), anti-JunB (C-11, Santa Cruz Biotechnology, 1:200), anti-BATF (D7C5, Cell Signaling Technology, 1:200), anti-IRF4 (IRF4.3E4, Biolegend, 1:200), anti-KLRG (2F1/KLRG1, Biolegend, 1:400), anti-CD127 (SB/199, Biolegend, 1:400), anti-Bim (C34C5, Cell Signaling Technology, 1:200), anti-Caspase-3 (C92-605, BD, 1:200), anti-programmed cell death 1 (PD-1) (RMP1-30, Biolegend, 1:400), anti-Tim3 (RMT3-23, Biolegend, 1:400), anti-Tigit (1G9, Biolegend, 1:400), anti-Lag-3 (C9B7W, Biolegend, 1:400), anti-CTLA4 (UC10-4B9, Biolegend, 1:400), anti-CD160 (7H1, Biolegend, 1:400), anti-TNF-α (MP6-XT22, Biolegend, 1:400), anti-GzmB (QA16A02, Biolegend, 1:400), and anti-IFN-γ (XMG1.2, Biolegend, 1:400). Anti-goat IgG (Poly4053, Biolegend, 1:200) and anti-rabbit IgG (Poly4064, Biolegend, 1:200) were used as isotype controls.

### Isolation of naive CD8^+^ T cells

Splenocytes were isolated from pooled spleens by mashing them through cell strainers (BD, 352340), followed by depleting non-CD8^+^ T cells using a Mojosort™ Mouse CD8^+^ T cell isolation Kit (Biolegend; 480008) according to the manufacturer’s instructions. These murine CD8^+^ T cells were then subjected to FACS of naive CD8^+^ T cells (CD8^+^ CD62L^hi^ CD44^lo^) using a BD FACS Aria II or Aria III. Sorting accuracy was 98%–99%.

### Adoptive transfer

Naive CD8^+^ T cells isolated from *Junb*^fl/fl^ or *Cd4*^cre^*Junb*^fl/fl^ OT-I mice (CD45.2^+^) were mixed with naive CD8^+^ T cells isolated from congenic OT-I mice (CD45.1^+^ CD45.2^+^) at a 1:1 ratio. These cells [1 × 10^4^ cells for most experiments, 1 × 10^5^ to 1 × 10^6^ cells for single-cell RNA-sequencing (scRNA-seq)] were intravenously injected into recipient B6SJL (CD45.1^+^) mice.

### Infection with Listeria monocytogenes

Mice were intravenously injected with 5 × 10^3^ colony-forming units (CFU) of LM-OVA (DMX, 09-082), a recombinant *Listeria monocytogenes* expressing ovalbumin (OVA) and an erythromycin-resistance gene, resuspended in 100 μl of phosphate-buffered saline (PBS). To prepare the LM-OVA inoculum, the bacterial frozen stock was streaked on a brain-heart infusion (BHI) (Sigma, 53286) agar plate and incubated overnight at 37°C. A single colony was picked from the plate, cultured in BHI media overnight, and then streaked on a BHI agar plate supplemented with erythromycin (10 μg/ml). A day before infection, an isolated colony of LM-OVA was cultured overnight in BHI media at 37°C with orbital shaking. Bacterial culture was diluted 25 times in BHI media and incubated for 2–3 h until reaching an OD_600_ of 0.1. Serial dilutions of the inoculum were plated on BHI agar plates to determine bacterial titers. The optical density (OD_600_) of 0.1 was estimated to correspond to 2 × 10^7^ CFU/ml.

### Cell isolation from the liver

Mice infected with LM-OVA were anesthetized and perfused with 50 ml of ice-cold PBS through the inferior vena cava while cutting the portal vein to drain the perfusate. The isolated liver was then subjected to enzymatic digestion using a liver dissociation kit (Miltenyi Biotec, 130-105-807). Isolated cells were filtered and resuspended into 37% Percol (Sigma, P1644), mixed with 70% Percol, and centrifuged for 10 min. Cells from the interface were isolated and used for flow cytometry analysis.

### Activation of OT-I T cells *in vitro*

Splenocytes isolated from B6SJL mice were irradiated with a dose of 2000 rads using an X-ray apparatus (Softx Co. Ltd, M-150WE). Subsequently, splenocytes were exposed to OVA peptides, N4 (SIINFEKL), T4 (SIITFEKL), or Q4H7 (SIIQFEHL) at a concentration of 100 nM. These peptides demonstrate high, intermediate, and low affinity for OT-I TCR, respectively. Splenocytes were washed and co-cultured with OT-I T cells for 24 h.

### Activation of polyclonal naive CD8^+^ T cells *in vitro*

Purified naive CD8^+^ T cells from *Junb*^*fl/fl*^ or *Cd4*^*cre*^*Junb*^*fl/fl*^ were cultured in 24-well (2 × 10^5^ cells per well), 48-well (1 × 10^5^ cells per well), or 96-well (0.5 × 10^5^ cells per well) non-treated culture plates coated with 5 mg/ml anti-CD3ε antibody (145-2C11, Biolegend) in Roswell Park Memorial Institute (RPMI) complete media (Invitrogen, 118785093) supplemented with 10% fetal bovine serum (FBS; Biosera, FB-1061), 100 U/ml penicillin, 100 mg/ml streptomycin (Sigma, P4333), 55 μM β-mercaptoethanol (Invitrogen, 20985-023), 10 nM 4-(2-hydroxyethyl)piperazine-1-ethanesulfonic acid (HEPES) (Invitrogen, 15630106), 1% of non-essential amino acids (Invitrogen, 11140050), and 1 mM sodium pyruvate (Invitrogen, 11360070). In addition, 1 mg/ml anti-CD28 antibody (37.51, Biolegend), 10 ng/ml IL-2 (Biolegend, 570402), and/or 10 ng/ml IL-12 (Biolegend, 577002) were added for *in vitro* CD8^+^ T cell activation. Activated cells were harvested for further analysis at the indicated time points. In some experiments, 5 nM c-Jun N terminal kinase (JNK) inhibitor II SP600125 (EMD Millipore, 420119), 5 nM rapamycin (Sigma, 553210), or 10 μM LY 2904002 PI3-kinase inhibitor (Invitrogen) were added.

### Flow cytometry

For analysis of cell surface molecules, cells were stained with fluorochrome-conjugated antibodies in PBS containing 2% FBS for 20 min on ice. OVA-specific CD8^+^ T cells were stained with H-2Kb-restricted OVA tetramer (MBL, TS-5001-1C). An SR-FLICA poly caspases assay kit (Immunochemistry technologies, IMT-916) was used to assess the total caspase activity. For analysis of intracellular molecules, cells were stained with fluorochrome-conjugated antibodies using a Foxp3 staining buffer set (eBioscience, 005253-00) according to the manufacturer’s instructions. For analysis of intracellular cytokines, cells were re-stimulated with phorbol 12-myristate 13-acetate (Sigma, P8139, 50 ng/ml) and ionomycin (Sigma, I0634, 500 ng/ml) in the presence of brefeldin A (Biolegend, 420601, 5 µg/ml). Before antibody staining, cells were incubated with anti-Fc receptor-blocking antibody (anti-CD16/CD32; Biolegend, 101320) and NIR-Zombie (Biolegend, 423106). The flow cytometry analyses were performed using a BD LSRFortessa™ X-20 cell analyzer or BD FACSAria™ III, and BD-FlowJo software was used to analyze the raw data. The gating strategies are shown in Supplementary Figure 1.

### scRNA-seq analysis

Cells were collected from spleens, quantified using FACS, and pooled in equal numbers from individual mice. Single-cell suspensions were loaded onto the 10X Genomics Chromium controller. Libraries were generated following the manufacturer’s protocol using a Chromium next GEM single cell 5ʹ v2 (dual index) reagent kit (10X Genomics). Reverse transcription was performed using a Bio-Rad T100 Thermal cycler. After assessing library quality using a Qubit fluorometer (Thermo Fisher), libraries were combined and sequenced on an Illumina Novaseq with a target of 20 000 reads per cell.

### scRNA-seq data analysis

Barcode processing, transcript counting, and alignment to the mm10 reference genome were performed using CellRanger v6.0.0 (10X Genomics) with default parameters. scRNA-seq data were analyzed using Seurat v4.1.0 R (38). Low-quality cells were filtered out based on mitochondrial gene counts (>7.5%) and numbers of expressed genes (<500) using the subset function. Then raw count of total RNA was normalized in each cell and rescaled across conditions using the NormalizeData function. Unsupervised clustering was performed on each dataset as follows: significant biological variation was captured using principal component analysis on top variant genes selected by FindVariableFeatures, followed by t-distributed stochastic neighborhood embedding (t-SNE) dimension reduction using the top 15 principal components. Cell clustering was performed with a resolution of 0.5 using the FindClusters function. Finally, marker genes were identified using the FindAllMarkers function with default parameters.

### Seahorse analysis

For extracellular flux assay, naive CD8^+^ T cells activated for 48 h were harvested and washed twice with PBS. Cells were then transferred to an analysis plate (2 × 10^5^ cells per well) coated with 25 μl of 2% gelatin (Sigma-Aldrich, G1890) and incubated at 37°C without CO_2_ for 1–2 h. A glycolysis stress kit (Agilent Technologies, 103015-100) and a Mito stress kit (Agilent Technologies, 103020-100) were used to measure the extracellular acidification rate (ECAR) and oxygen consumption rate (OCR), respectively, with a Seahorse XFe96 analyzer (Seahorse Bioscience, Agilent Technologies). For ECAR analysis, cells were incubated in XF base media containing 2 mM glutamine (Gibco, A2916801) and treated with 10 mM glucose (Gibco; A2494001), 1 µM oligomycin, and 50 mM 2-deoxyglucose (2-DG). For OCR analysis, cells were cultured in XF base media containing 1 mM pyruvate (Gibco, 11360070), 2 mM glutamine, and 10 mM glucose and treated with 1.5 µM oligomycin, 1 µM fluorocarbon cyanide phenylhydrazone, and 0.5 µM rotenone/antimycin A.

### Bulk RNA-seq

Naive CD8^+^ T cells activated by anti-CD3 and anti-CD28 antibodies, IL-2, and IL-12 for 96 h were stained with zombie-NIR (Biolegend, 423105, 1:400), and viable cells (zombie-NIR negative) were sorted using FACS. RNA was extracted from the sorted cells using Trizol (Invitrogen) and Qiagen RNAeasy mini kit (Qiagen). cDNA libraries were prepared using a NEBNext Ultra II directional RNA library prep kit for Illumina (New England Biolabs, E7760L) and purified using Agentcourt AMPure XP beads (Beckman Coulter, A63880). Adapter dimers in cDNA libraries were removed using a LabChip NGS 3K reagent kit (PerkinElmer, CLS960013), and library quality was verified using a tape station (Agilent). Quantification of cDNA libraries was performed using digital droplet PCR with the BioRad QX-200 system. Samples were sequenced using an Illumina NovaSeq 6000, generating 150-nucleotide, paired-end reads with a minimum read depth of at least 20 million reads per sample.

### Bulk RNA-seq data analysis

RNA-seq data were processed using Cutadapt 2.10 to remove adaptors and low-quality sequences (the cutoff of the *Q*-score is 25). Data were then aligned to the UC Santa Cruz (UCSC) mouse genome mm10, and transcripts were quantified using Salmon 1.3.0 with default settings. A mouse genome index with a *k* value of 31 was used for gene annotation (39). Following transcript quantification, transcript counts within and between samples were normalized to obtain transcripts per kilobase million. Differential gene expression analysis was performed using DESeq2 (40). Genes differentially expressed between *Junb*-deficient and control cells (with a log_2_ fold change <−0.5 or >0.5, *P* value <.05, and base mean >25) were used for pathway analysis using Enrichr (41).

### Assay for transposase-accessible chromatin sequencing (ATAC-seq)

ATAC sample preparation and sequencing were performed according to a previously described protocol (42) at the OIST sequencing section. Briefly, FACS-sorted living cells (5 × 10^4^ per sample) were snap-frozen in liquid nitrogen, thawed, and lysed in a buffer (10 mM Tris–HCl, 10 mM NaCl, 3 mM MgCl_2_, 0.1% NP40, 0.1% Tween20, and 0.01% Digitonin). Nuclei were collected and subjected to DNA tagmentation using a Tagment DNA Kit (Illumina, 20034210). DNA was purified using a Zymo DNA Clean and Concentrator 5 kit (Zymo, D4014) and used for PCR amplification and oligonucleotide indexing with NEBNext High-Fidelity 2X PCR Master Mix (New England Biolabs, M0541S). Subsequently, DNA was purified twice using solid-phase reversible immobilization (SPRI) beads (Beckman Coulter, B23318) with a 0.5–1 ratio of SPRI beads to DNA in the first purification and 1.3–1 in the second purification. Sequencing library DNA was quantified using a Qubit dsDNA HS Assay kit (Invitrogen, Q32851), and its size distribution was assessed using an Agilent High sensitivity DNA kit (Agilent, 5067-4626). Samples were sequenced on an Illumina Novaseq 6000 sequencer, generating paired-end reads of 150 base pairs in length with a depth of at least 20 million reads per sample.

### ATAC-seq data analysis

ATAC-seq fastq reads were trimmed using Trimmomatic v0.39. with arguments SLIDINGWINDOW420MINLEN:35 LEADING:20 TRAILING:20 (43). Subsequently, sequence quality was evaluated with FASTQC v0.11.9, and duplicates were removed using the MarkDuplicates function from Picard v2.7.0 (44). Reads were then mapped to genomic regions listed in the ENCODE mm10 blacklist. Read positions were then corrected with a constant offset to the read start (plus strands: +4 bp, - strands: −5 bp) with deepTools v.3.5.1 using the “alignmentSieve-ATACshift” option. Peak calling was performed using MACS2 v2.2.7.1 to obtain narrow peaks with the “-fBAMP—nomode--shift 75--extsize 150” option (45). BigWig files were created with bamCoverage from deepTools and were uploaded to the UCSC genome browser (46). Differential peak analysis was conducted with the Diffbind package. ChIPseeker was used for peak annotation (47). Motif enrichment in the differentially accessible chromatin regions (DACRs) was analyzed using the findMotifs function of Homer (version v4.11).

### Statistical analysis

Unpaired two-tailed Student’s tests and one-way analysis of variance followed by Tukey’s *post hoc* tests were performed with Prism (GraphPad). *P* values <.05 were considered statistically significant.

### Data availability

Processed scRNA-seq data have been deposited in the Zenodo Repository (DOI: 10.5281/zenodo.10558743). Bulk-RNA-seq and ATAC-seq data are available at PRJDB17429.

## Results

### Induction of JunB expression in CD8^+^ T cell responses

**Figure 1.**
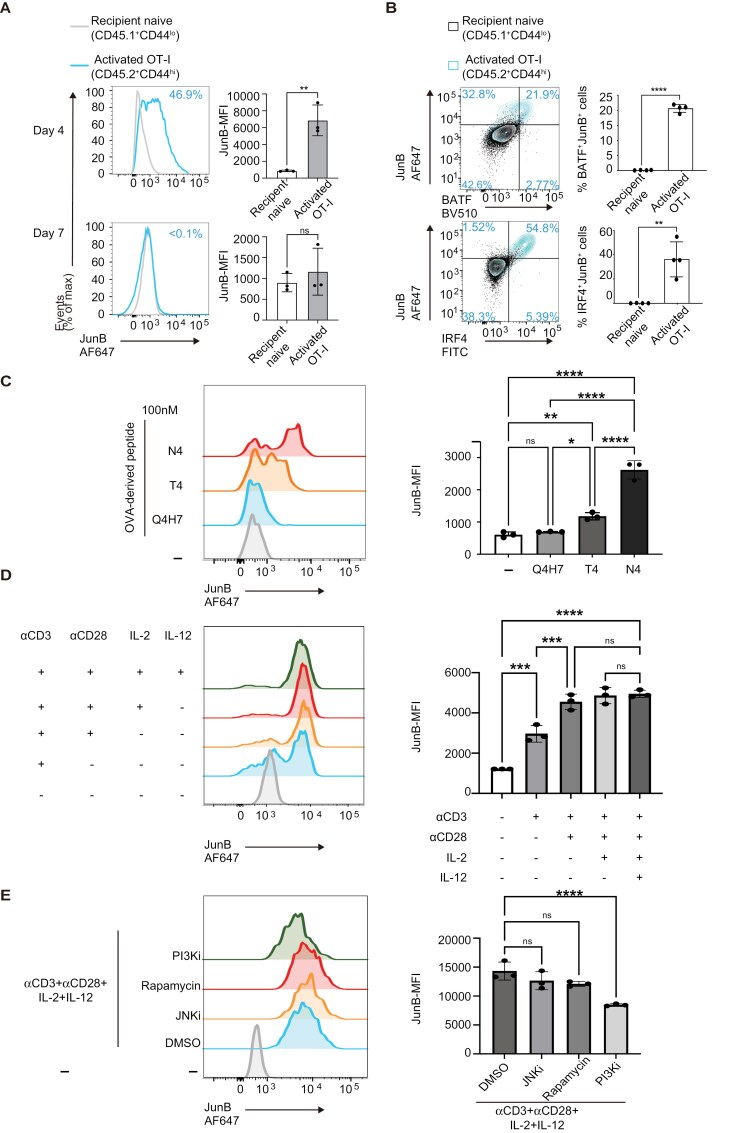
JunB expression is induced in CD8^+^ T cells activated in response to *L. monocytogenes* infection. (A, B) OT-I T cells (CD45.2^+^) were transferred into congenic recipient mice (CD45.1^+^), followed by LM-OVA infection. Cells isolated from the spleen on Days 4 and 7 p.i. were subjected to flow cytometry analysis. (A) Left: representative histograms showing expression of JunB in activated OT-I T cells (CD45.2^+^ CD44^hi^) or recipient naive CD8^+^ T cells (CD45.1^+^ CD44^lo^). Right: graph showing MFI of JunB expression. (B) Left: plots showing expression of JunB and BATF, or JunB and IRF4 in OT-I T cells gated on CD45.2^+^ and that in recipient CD8^+^ T cells gated on CD45.2^−^. Right: graph showing percentages of cells co-expressing JunB and BATF or IRF4. Error bars indicate 1 SD (*n* = 3–4). *P < .01, ***P < .001, ****P < .0001 (unpaired two-tailed Student’s test). Data are representative of two independent experiments. (C) OT-I T cells were stimulated with irradiated splenocytes pulsed with an OVA-derived peptide (N4) or its variants (T4 or Q4H7). At 24 h after activation, JunB expression was analyzed by flow cytometry. (D) Naive CD8^+^ T cells were activated by anti-CD3 antibody with or without anti-CD28 antibody in the presence or absence of cytokines IL-2 or IL-12. At 24 h after activation, JunB expression was analyzed by flow cytometry. (E) Naive CD8^+^ T cells were activated by anti-CD3 antibody with or without anti-CD28 antibody in the presence of pharmacological inhibitors for JNK (JNKi), PI3K (PI3Ki), or mTOR (rapamycin). At 24 h post activation, JunB expression was analyzed by flow cytometry. (C–E) Left: flow cytometry histograms showing JunB expression. Right: graph showing mean fluorescence intensity (MFI) of JunB expression. Error bars indicate SD (*n* = 3). **P < .01, ***P < .001, ****P < .0001, ns: not significant (one-way ANOVA with Bonferroni’s multiple comparison tests). Data are representative of two independent experiments. ANOVA, analysis of variance.

To analyze JunB expression during CD8^+^ T cell responses to acute infections, we adoptively transferred naive CD8^+^ OVA-specific transgenic (OT-I) T cells into congenic recipient mice, followed by infection with *L. monocytogenes* expressing OVA antigen (LM-OVA). In this acute infection model, OT-I T cells undergo clonal expansion, reaching peak levels by approximately Day 7 post-infection (p.i.), after which they contract and generate memory T cells (48). Flow cytometry analysis showed that JunB was expressed in approximately half of activated OT-I T cells exhibiting a CD44^hi^ phenotype on Day 4 p.i., while its expression was not detectable in host naive CD8^+^ T cells with a CD44^lo^ phenotype (Fig. 1A). Subsequently, JunB expression declined and was no longer detectable by Day 7 p.i. (Fig. 1A). In JunB-expressing OT-I T cells, BATF and IRF4 were also expressed (Fig. 1B and Supplementary Figure 2A and B). Thus, expression of JunB, along with BATF and IRF4, is transiently induced during clonal expansion of CD8^+^ T cells in response to acute infection.

TCR signal strength influences expression levels of BATF and IRF4 (29, 30). To assess whether TCR signal strength also affects JunB expression, we activated OT-I T cells with an OVA-derived peptide SIINFEKL (N4) or its variants, SIITFEFL (T4) or SIIQFEHL (Q4H7) *in vitro*. These peptides presented by major histocompatibility complex (MHC) class I molecules show different affinities to OT-I TCR (N4 > T4 > Q4H7) (49). Q4H7 did not induce JunB expression, and T4 induced low levels of JunB expression, whereas N4 induced significantly high levels of JunB expression (Fig. 1C). A similar trend was observed in IRF4 expression, as previously reported (29, 30), while BATF expression was similarly induced in cells stimulated with T4 and N4, but not Q4H7 (Supplementary Figure 2C). We also found that JunB deficiency significantly reduced expression of IRF4, but not BATF, in cells stimulated with N4 (Supplementary Figure 2C). These results imply that levels of JunB expression induced in activated T cells depend on TCR signal strength in a manner similar to IRF4.

TCR stimulation (Signal 1), CD28 co-stimulation (Signal 2), and cytokine signals such as IL-2 and IL-12 (Signal 3) are required for the differentiation of naive CD8^+^ T cells to effector CD8^+^ T cells (2). Accordingly, we next assessed the impacts of the co-stimulatory signal and inflammatory cytokines on JunB expression in TCR-stimulated CD8^+^ T cells. This revealed that JunB expression induced by anti-CD3 antibody stimulation was promoted by co-stimulation with anti-CD28 antibody (Fig. 1D). However, addition of cytokines, IL-2 and IL-12, did not further enhance JunB expression induced by anti-CD3 and anti-CD28 antibodies (Fig. 1D). In contrast, expression of BATF and IRF4 was induced by anti-CD3 antibody, and it was slightly enhanced by IL-12, but not anti-CD28 antibody (Supplementary Figure 2D). We also found that JunB deficiency significantly decreased expression of IRF4, but not BATF, in cells stimulated with anti-CD3 and anti-CD28 antibodies, IL-2, and IL-12 (Supplementary Figure 2D).

The phosphatidylinositol 3-kinase (PI3K)/Akt signaling pathway, JNK pathway, and mammalian target of rapamycin (mTOR) pathway are involved in TCR and the CD28 co-stimulatory signal transduction (50). Therefore, we next examined whether these pathways mediate JunB expression in CD8^+^ T cell activation using their pharmacological inhibitors. This revealed that induction of JunB expression during CD8^+^ T cell activation was impaired by inhibition of the PI3K pathway, whereas inhibition of the JNK or mTOR pathway did not affect JunB expression (Fig. 1E). Inhibition of the PI3K pathway also reduced expression of BATF and IRF4 (Supplementary Figure 2E). As previously reported (29), we also observed that inhibition of the mTOR pathway inhibited IRF4 expression (Supplementary Figure 2E). These data indicate that JunB expression is induced upon activation of naive CD8^+^ T cells in a manner dependent on TCR and CD28 co-stimulatory signaling.

### JunB is required for clonal expansion of effector CD8^+^ T cells in response to acute infection

**Figure 2.**
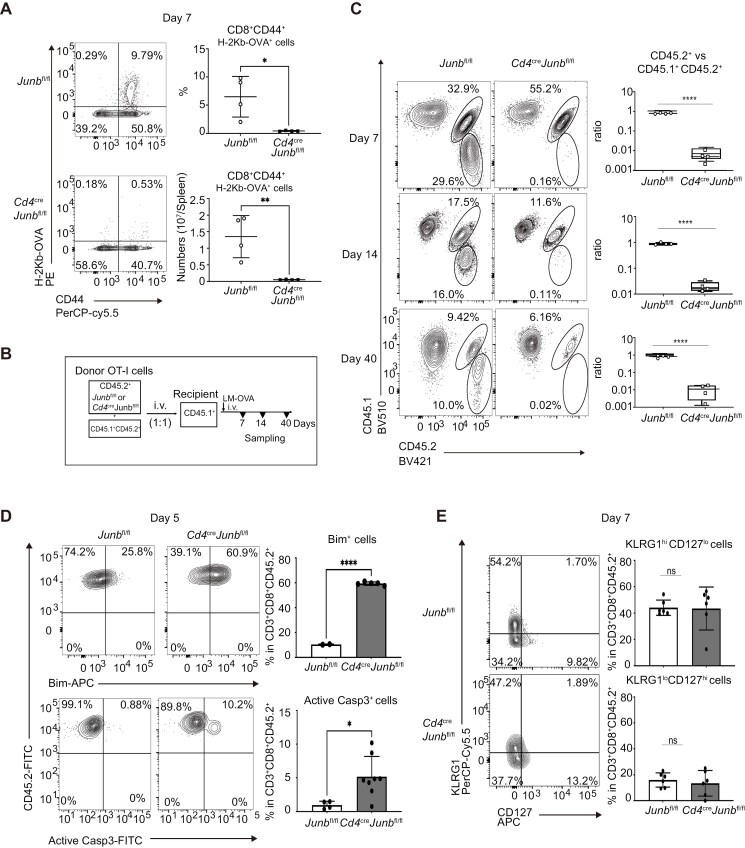
JunB is required for clonal expansion of CD8^+^ T cells in response to *L. monocytogenes* infection. (A) Control (*Junb*^fl/fl^) or *Junb*-deficient (*Junb*^fl/fl^*Cd4*^Cre^) mice were infected with LM-OVA (5 × 10^3^ CFU). On Day 7, cells were isolated from the spleen and subjected to flow cytometry analysis of binding to OVA tetramer. Left: representative plots showing binding of OVA tetramers (H-2Kb-restricted OVA tetramer) and expression of CD44 in cells gated on CD3^+^ CD8^+^. Right: graphs showing proportions of H-2Kb-restricted OVA tetramer^+^ CD44^+^ cells in CD8^+^ T cells (top) and their absolute numbers (bottom). Error bars indicate SD (*n* = 4 per group). (B–E) Control (*Junb*^fl/fl^) or *Junb*-deficient (*Junb*^fl/fl^*Cd4*^Cre^) OT-I T cells (CD45.1^−^ CD45.2^+^) (1 × 10^4^ cells) were transferred with an equal number of wild-type OT-I T cells (CD45.1^+^ CD45.2^+^) into congenic recipient mice (CD45.1^+^ CD45.2^−^), followed by infection with LM-OVA. At the indicated time points, cells were isolated from the spleen and subjected to flow cytometry analysis. (B) Schematic showing the experimental design. (C) Flow cytometry analysis of the frequency of OT-I T cells (gated on CD3^+^CD8^+^ T cells) on Days 7, 14, and 40. Left: representative plots showing expression of CD45.1 and CD45.2. Numbers next to the outlined areas indicate the percentage of cells expressing the surface markers. Right: graphs showing the ratio of *Junb*^fl/fl^ or *Cd4*^cre^*Junb*^fl/fl^ OT-I T cells (CD45.1^−^ CD45.2^+^) versus co-transferred OT-I T cells (CD45.1^+^CD45.2^+^). Error bars indicate SD (*n* = 4 per group). (D) Flow cytometry analysis of expression of Bim and active Caspase 3 in OT-I T cells on Day 5. Left: representative plots showing expression of Bim (top) or active Caspase 3 (bottom). Right: graphs showing percentages of OT-I T cells expressing Bim (top) or active Caspase 3 (bottom). Error bars indicate SD (*n* = 3–6 per group). (E) Flow cytometry analysis of expression of KLRG1 and CD127 in OT-I T cells on Day 7. Left: representative plots. Right: graphs showing percentages of SLECs (KLRG1^hi^CD127^lo^) and MPECs (KLRG1^lo^CD127^hi^). Error bars indicate SD (*n* = 3–4 per group). (A, C–E) *P < .05, **P < .01, ****P < .0001 (unpaired two-tailed Student’s *t*-test). Data represent two independent experiments.

To determine whether JunB is involved in CD8^+^ T cell responses to acute infection, we infected T-cell-specific *Junb*-deficient (*Cd4*^*cre*^*Junb*^*fl/fl*^) and control (*Junb*^*fl/fl*^) mice with LM-OVA and quantified OVA-specific T cells using an H-2Kb-restricted OVA tetramer. Substantial numbers of OVA-specific CD8^+^ T cells were detected in spleens and livers in control mice but not in *Junb*-deficient mice on Days 7 and 14 p.i. (Fig. 2A and Supplementary Figure 3A–C). To further prove the importance of CD8^+^ T cell-intrinsic JunB expression, we adoptively transferred *Junb*-deficient or control OT-I T cells (CD45.2^+^) in combination with equal numbers of congenic OT-I T cells (CD45.1^+^CD45.2^+^) into CD45.1^+^ recipient mice and then infected with LM-OVA (Fig. 2B). Control OT-I T cells and co-transferred congenic cells exhibited comparable cell numbers, with a significant increase until Day 7 p.i., followed by a decline, yet a substantial number of cells persisted even on Day 40 p.i. (Fig. 2C). In contrast, the frequency of *Junb*-deficient OT-I T cells was over 100-fold lower than that of co-transferred congenic cells at all time points (Fig. 2C).

In the differentiation of CD4^+^ helper T cells or effector Treg cells, JunB inhibits apoptosis by repressing the expression of *Bcl2l11* (encoding Bim) to support cell survival (36, 37). Accordingly, we examined the expression of Bim and an active form of Caspase 3 in *Junb*-deficient OT-I T cells in mice infected with LM-OVA. On Day 5 p.i., there was a significant increase in the expression of Bim and active Caspase 3 in *Junb*-deficient OT-I T cells compared with control cells (Fig. 2D). We also investigated whether JunB controls the proportions of SLECs and MPECs by analyzing the expression of KLRG1 and CD127 in a few *Junb*-deficient OT-I T cells detected on Days 7 and 14 p.i. We observed comparable ratios of SLECs (KLRG1^hi^CD127^lo^) to MPECs (KLRG1^lo^CD127^hi^) between control and *Junb*-deficient OT-I T cells at both time points (Fig. 2E and Supplementary Figure 3D). These results suggest that intrinsic JunB inhibits apoptosis to support clonal expansion of effector CD8^+^ T cells in response to acute infection and is required for accumulation of both SLECs and MPECs.

### JunB promotes the generation of proliferative effector CD8^+^ T cells in response to acute infection

**Figure 3.**
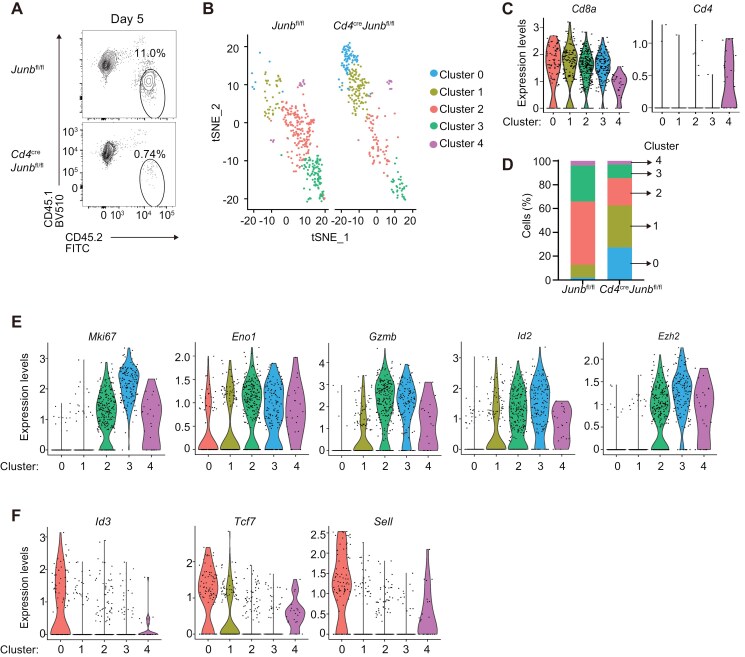
JunB is critical to generate proliferating effector CD8^+^ T cells. Control (*Junb*^fl/fl^) or *Junb*-deficient (*Junb*^fl/fl^*Cd4*^Cre^) OT-I T cells (CD45.2^+^) were transferred into congenic recipient mice (CD45.1^+^), followed by infection with LM-OVA. On Day 5, living OT-I T cells (CD45.2^+^) were sorted from splenocytes and subjected to scRNA-seq analysis. t-SNE clustering analysis was performed using pooled scRNA-seq data of control and *Junb*-deficient OT-I T cells. (A) Flow cytometry plots showing the frequency of OT-I T cells (CD45.2^+^) before sorting. (B) t-SNE clustering detected five clusters (Clusters 0–4). (C) Violin plots showing *Cd8a* and *Cd4* expression in each cluster. (D) Stacked bar charts showing percentages of cells in each cluster. Violin plots showing expression of representative genes highly expressed in Clusters 2 and 3 (E) and those highly expressed in Clusters 0 and/or 1 (F). Each gene name is shown on the plots.

To further investigate how JunB promotes clonal expansion of effector CD8^+^ T cells, we next evaluated the impact of JunB deficiency on OT-I T cells during clonal expansion by scRNA-seq analysis. For this, mice transferred with control or *Junb*-deficient OT-I T cells were infected with LM-OVA. On Day 5 p.i., a few days before the peak of clonal expansion, we harvested splenocytes and purified OT-I T cells using FACS. Already at this time point, the frequency of *Junb*-deficient OT-I T cells was significantly lower than controls (Fig. 3A), but we obtained enough cells for scRNA-seq analysis. Unsupervised clustering and t-SNE visualization of scRNA-seq data revealed that control and *Junb*-deficient OT-I T cells were classified into four clusters (Clusters 0–3) comprising cells highly expressing *Cd8a* along with a smaller cluster (Cluster 4) characterized by expression of moderate levels of both *Cd8a* and *Cd4* (Fig. 3B and C). Control cells were predominantly found in Clusters 2 and 3, whereas *Junb*-deficient cells were mainly found in Clusters 0 and 1 (Fig. 3B and D). Cells in Clusters 2 and 3 appeared to be proliferative effector cells because of their elevated expression of genes related to cell proliferation (*Mki67*, *Pcna*, and *Top2a*), glycolysis (*Phgdh* and *Eno1*), cytotoxic T lymphocyte (CTL) effector functions (*Gzma* and *Gzmb*), and SLEC differentiation (*Id2*, *Tbx21*, *Klrg1*, and *Ezh2*) (Fig. 3E and Supplementary Figure 4A). In contrast, many cells in Cluster 0 and some cells in Cluster 1 exhibited high expression of genes associated with naive or memory CD8^+^ T cells, such as *Id3*, *Tcf7*, *Lef1*, *Sell*, *Cd27*, and *Slamf6* (Fig. 3F and Supplementary Figure 4B). Accordingly, we observed that *Junb*-deficient cells tended to exhibit a lower frequency of cells expressing *Id2* or *Tbx21* and a higher frequency of cells expressing *Id3* or *Tcf7* compared with controls (Supplementary Figure 4C). These results suggest that JunB may promote differentiation of proliferative effector CD8^+^ T cells in the early phase of response to acute infection.

### JunB promotes survival and glycolysis in effector CD8^+^ T cell differentiation

**Figure 4.**
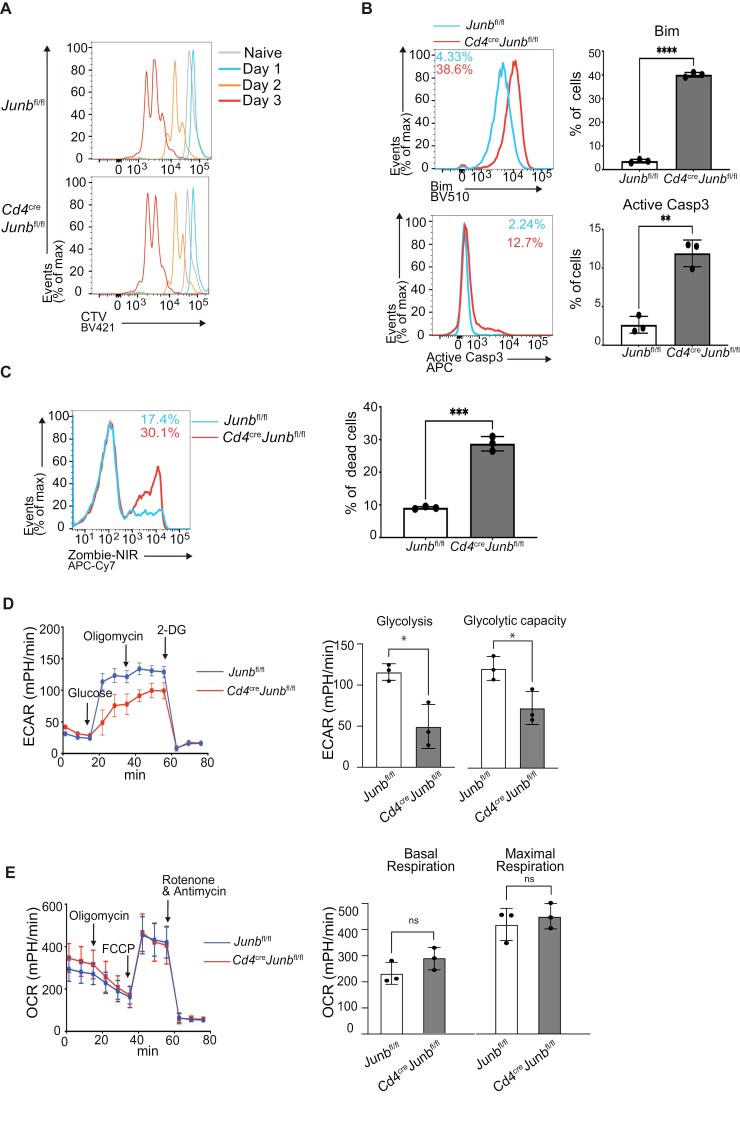
JunB promotes survival of naive CD8^+^ T cells activated with strong stimulatory signals *in vitro*. Control (*Junb*^fl/fl^) or *Junb*-deficient (*Junb*^fl/fl^*Cd4*^Cre^) naive CD8^+^ T cells were activated with anti-CD3, anti-CD28, IL-2, and IL-12 *in vitro*. (A) Cell proliferation was analyzed by flow cytometry. Prior to activation, naive CD8^+^ T cells were stained with cell trace violet (CTV), and CTV dilution was analyzed on Days 1, 2, and 3 after activation. (B) Expression of Bim and active Caspase 3 at 48 h after activation was analyzed by flow cytometry. Left: representative histograms. Right: graphs showing percentages of cells expressing Bim (upper panel) and active Caspase 3 (lower panel). (C) Cell viability at 72 h after activation was analyzed by flow cytometry of cells stained with Zombie-NIR dye. Left: representative histograms. Right: graph showing percentages of dead cells stained with Zombie-NIR. (D) ECAR was measured using seahorse analysis. Cells activated for 48 h were placed on a Seahorse assay plate and were incubated with sequential injections of glucose, oligomycin, and 2-DG. Left: kinetics of ECAR. Right: graphs showing ECAR in basal glycolysis and glycolytic capacity. (E) OCR was measured using seahorse analysis. Cells activated for 48 h were placed on a Seahorse assay plate and were incubated with sequential injections of oligomycin, FCCP, and rotenone/antimycin. Left: kinetics of OCR. Right: graphs showing OCR in basal and maximal respiration. (B–E) Error bars indicate SD (*n* = 3). *P < .05, **P < .01, ***P < .001, ****P < .0001 (unpaired two-tailed Student’s *t*-test). Data are representative of two independent experiments. FCCP, fluorocarbon cyanide phenylhydrazone.

To further investigate the role of JunB in CD8^+^ T cell responses, we used an *in vitro* model of activation of naive CD8^+^ T cells, because this allows evaluation of the effect of JunB deficiency on cell populations at specific differentiation stages. Considering our *in vivo* findings, we first assessed whether JunB is involved in the regulation of apoptosis, cell proliferation, and glycolysis in naive CD8^+^ T cells stimulated with anti-CD3 and anti-CD28 antibodies in the presence of IL-2 and IL-12. A cell trace violet dilution assay showed comparable cell division between *Junb*-deficient CD8^+^ T cells and controls (Fig. 4A). On the other hand, flow cytometry analysis revealed that *Junb*-deficient CD8^+^ T cells exhibited enhanced expression of Bim and active Caspase 3 at 48 h post-stimulation and an increase in frequency of cells stained with cell death marker, zombie-NIR, at 72 h (Fig. 4B and C). Additionally, using *Junb*-deficient CD8^+^ T cells activated for 48 h, at which their viability was still comparable to controls, we examined levels of glycolysis and oxidative phosphorylation. This revealed that JunB deficiency significantly reduced the ECAR, an indicator of glycolysis (Fig. 4D). In contrast, it did not affect the OCR, an indicator of oxidative phosphorylation (Fig. 4E). These data confirm that JunB inhibits apoptosis and promotes metabolic reprogramming of glycolysis in strongly activated CD8^+^ T cells.

### JunB controls the expression of genes associated with CD8^+^ T cell responses

**Figure 5.**
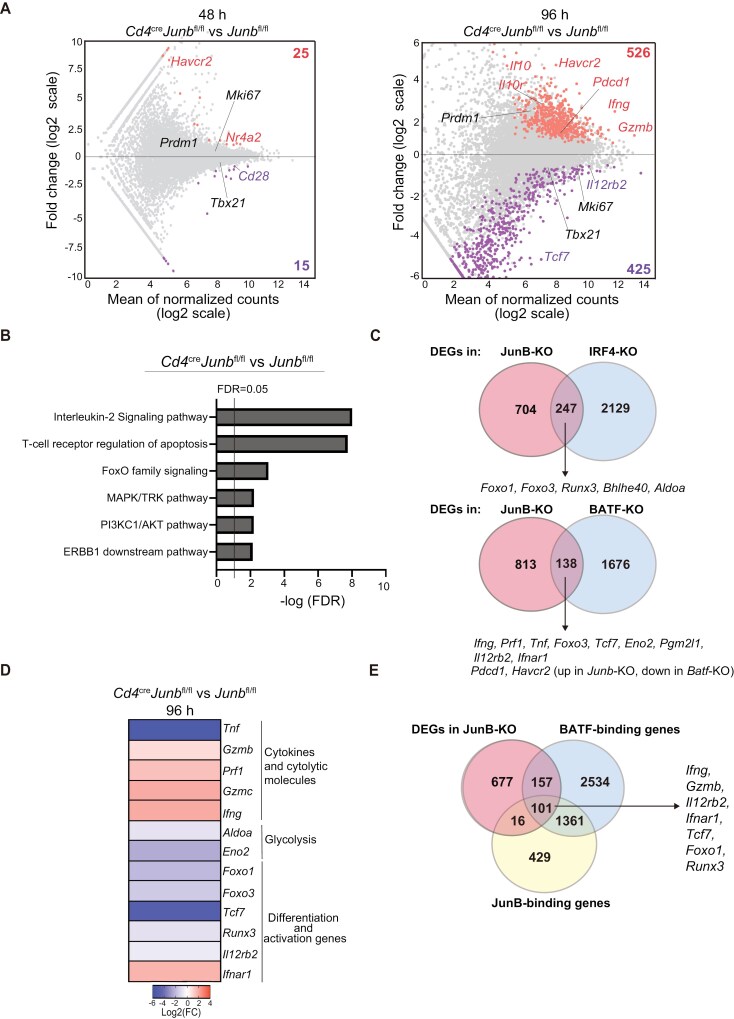
JunB regulates the expression of genes associated with CD8^+^ T cell responses. Control (*Junb*^fl/fl^) or *Junb*-deficient (*Junb*^fl/fl^*Cd4*^Cre^) naive CD8^+^ T cells were activated by anti-CD3 and anti-CD28 antibodies, IL-2, and IL-12 *in vitro* for 48 or 96 h and subjected to RNA-seq analysis (*n* = 3–4). (A) Mean average plots of DEGs between control and *Junb*-deficient cells (log_2_ fold change >0.5 or <−0.5, adjusted *P* value <.05). Significantly upregulated and downregulated genes in *Junb*-deficient cells are marked. DEG numbers and names of representative genes are shown; genes downregulated by JunB deficiency are shown in purple, upregulated genes in orange, and unchanged genes in black. (B) Enrichr pathway analysis of DEGs at 96 h. (C) Venn diagrams show the relationship between genes regulated by JunB and IRF4 (upper panel) and BATF (lower panel). DEGs in *Junb*-deficient CD8^+^ T cells were compared with genes affected by deficiency of BATF and IRF4 (obtained from previous studies, GSE49929, GSE54215). (D) Heat map showing DEGs related to cytokines and cytolytic molecules, genes related to glycolysis, and differentiation/activation of effector and memory CD8^+^ T cells. (E) Venn diagrams showing DEGs in *Junb*-deficient CD8^+^ T cells, categorized by whether they were bound by JunB or BATF.

To understand the role of JunB in transcriptional regulation of effector CD8^+^ T cell differentiation, we performed bulk RNA-seq analysis of control and *Junb*-deficient naive CD8^+^ T cells activated *in vitro* for 48 or 96 h. This analysis detected 40 differentially expressed genes (DEGs, log_2_ fold change >0.5, adjusted *P* values <.05) between *Junb*-deficient and control cells at 48 h after activation and 951 DEGs at 96 h (Fig. 5A). DEGs upregulated by JunB deficiency included *Ifng*, *Granzyme b* (*Gzmb*), *Nr4a2*, *Interleukin 10* (*Il10*), *Il10 receptor* (*Il10r*), *Programmed cell death 1* (*Pdcd1* encoding PD-1), and *Hepatitis A virus cellular receptor 2* [*Havcr2* encoding T cell immunoglobulin and mucin domain-3 (TIM3)], while those downregulated included *Cd28*, *Il12rb2*, and *Tcf7* (encoding Tcf1) (Fig. 5A). The majority of these genes were identified at either time point, but *Havcr2* was detected at both time points (Fig. 5A). Pathway enrichment analysis of DEGs revealed that JunB deficiency significantly affects the pathways related to *IL-2 signaling* and *T cell receptor regulation of apoptosis* (Fig 5B).

We next compared DEGs in *Junb*-deficient CD8^+^ T cells with those identified in previous transcriptomic analyses of *Batf*- or *Irf4*-deficient CD8^+^ T cells activated *in vitro* (20, 30). This revealed that 14.5% (247 out of 951) and 25.9% (138 out of 951) of DEGs in *Junb*-deficient CD8^+^ T cells overlapped with DEGs in *Batf*- and *Irf4*-deficient CD8^+^ T cells, respectively (Fig. 5C). We found that JunB deficiency affected the expression of several key genes controlled by BATF and IRF4. For example, as reported for BATF (20), JunB deficiency decreased the expression of tumor necrosis factor a (*Tnfa*), while increasing the expression of *Ifng* and *Gzmb* (Fig. 5C and D). Flow cytometry analysis confirmed differential expression of these cytokines in *Junb*-deficient CD8^+^ T and controls (Supplementary Figure 5). Furthermore, consistent with our *in vivo* observations, JunB deficiency resulted in reduced expression of genes related to glycolysis, *enolase 2* (*Eno2*) and *aldolase A* (*Aldoa*), whose expression was also affected by deficiency of BATF or IRF4 (Fig. 5C and D). Additionally, like deficiency of BATF or IRF4, JunB deficiency decreased the expression of genes associated with T cell activation and effector/memory cell differentiation, such as *Foxo1*, *Foxo3*, *Runx3*, *Tcf7*, *Il12rb2*, and *Ifnar1* (Fig. 5C and D). However, we noted that unlike BATF and IRF4 (20, 29), JunB deficiency did not affect the expression of *Tbx21* and *Prdm1* (Fig. 5A). Additionally, consistent with our observation that proliferation of *Junb*-deficient CD8^+^ T cells activated *in vitro* was normal (Fig. 4A), JunB deficiency did not affect the expression of *Mki67* proliferation marker gene (Fig. 5A).

We next examined whether JunB, collaborating with BATF, regulates the expression of its target genes by directly binding the loci or indirectly, using chromatin immunoprecipitation sequencing (ChIP-seq) data from the database (20). This revealed that JunB, together with BATF, bound to 10.6% (101 out of 951) of the loci of DEGs identified in *Junb*-deficient CD8^+^ T cells (Fig. 5E). Collectively, these results suggest that JunB, in collaboration with BATF and IRF4, controls expression of a subset of genes important for effector CD8^+^ T cell responses.

### JunB suppresses expression of co-inhibitory molecules

**Figure 6.**
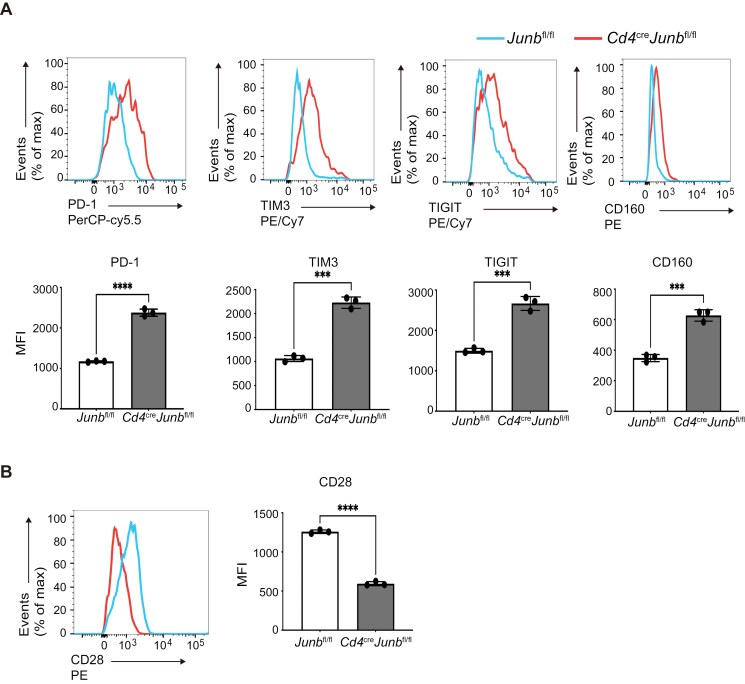
JunB controls the expression of co-inhibitory and co-stimulatory receptors. Naive Control (*Junb*^fl/fl^) or *Junb*-deficient (*Junb*^fl/fl^*Cd4*^Cre^) naive CD8^+^ T cells were activated by anti-CD3 and anti-CD28 antibodies, IL-2, and IL-12 *in vitro* for 96 h. Flow cytometry analysis of co-inhibitory receptors, PD-1, TIM3, TIGIT, and CD160 (A), and co-stimulatory receptor CD28 (B). Error bars indicate SD (*n* = 3). ***P < .001, ****P < .0001 (unpaired two-tailed Student’s *t*-test). Data are representative of two independent experiments.

PD-1, a co-inhibitory molecule closely associated with T cell exhaustion (51, 52), is also expressed during the early stage of effector CD8^+^ T cell differentiation and negatively regulates the differentiation process (53). In the above RNA-seq analysis, we found that JunB deficiency significantly increased mRNA expression of *pdcd1* (encoding PD-1) and another co-inhibitory receptor, *Havcr2* in effector CD8^+^ T cell differentiation (Fig. 5A). Conversely, BATF deficiency decreased the expression of these genes (Fig. 5C) (20). To extend these observations, we analyzed whether JunB deficiency affects the protein expression of various co-inhibitory molecules during effector CD8^+^ T cell differentiation. Flow cytometry analysis showed that JunB deficiency increased the expression of PD-1, TIM3, T cell immunoreceptors with Ig and ITIM domains (TIGIT), and CD160 (Fig. 6A). We also found that JunB deficiency reduced the expression of a co-stimulatory molecule, CD28 (Fig. 6B). We also conducted flow cytometry analysis of OT-I T cells isolated from LM-OVA-infected mice at Day 5 post-infection. *Junb*-deficient OT-I T cells showed a slight but statistically significant increase in expression of PD-1 but not Tim3 (Supplementary Figure 6A and B). These results indicate that JunB is required for proper expression of co-stimulatory and co-inhibitory molecules during effector CD8^+^ T cell differentiation.

### JunB regulates chromatin accessibility of target genes in effector CD8^+^ T cell differentiation

**Figure 7.**
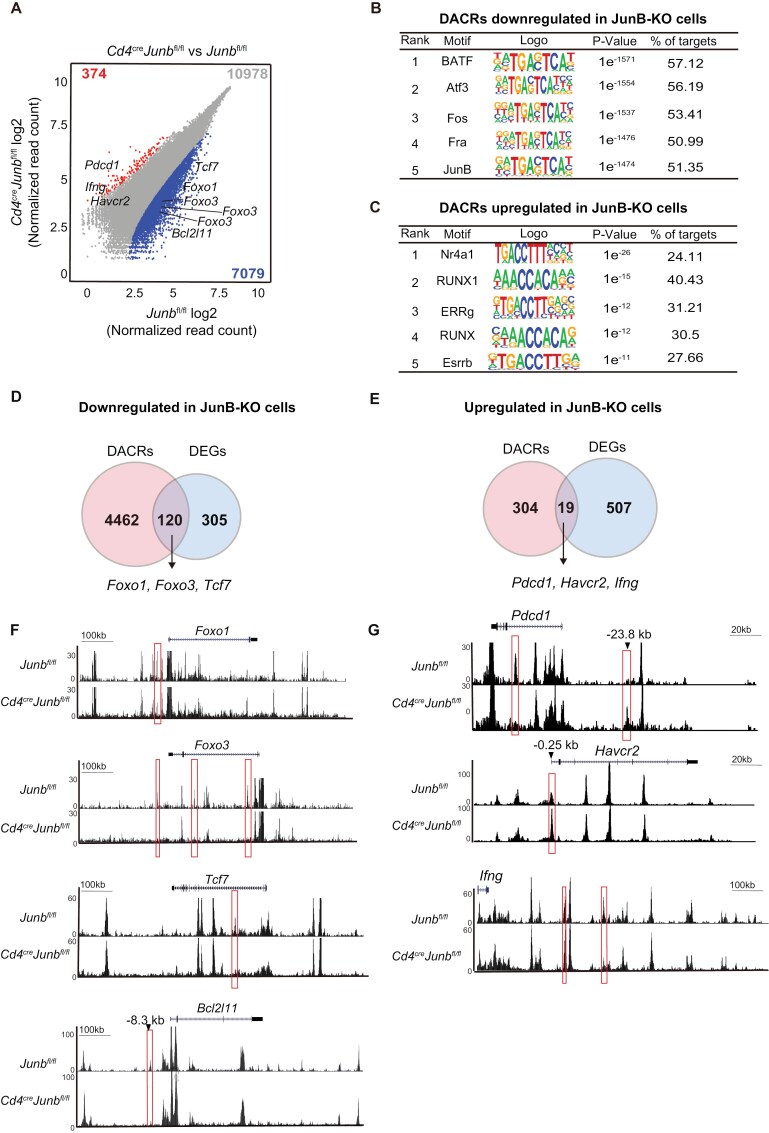
JunB governs chromatin accessibility to regulate a subset of transcriptional regulatory target genes. Control (*Junb*^fl/fl^) or *Junb*-deficient (*Junb*^fl/fl^*Cd4*^Cre^) naive CD8^+^ T cells were activated by anti-CD3 and anti-CD28 antibodies, IL-2, and IL-12 *in vitro* for 96 h and subjected to ATAC-seq analysis. (A) Scatterplot showing DACRs in *Junb*-deficient CD8^+^ T cells vs control cells (log_2_ fold change >0.5, false discover rate <0.05). Chromatin-accessible regions with decreased accessibility and increased accessibility in *Junb-*deficient cells are marked. Numbers on the plot represent total counts of chromatin-accessible regions (in gray), along with those exhibiting decreased accessibility (in blue) and increased accessibility (in red) in *Junb-*deficient CD8^+^ cells. Motif enrichment analysis of DACRs with decreased (B) or increased (C) accessibility in *Junb*-deficient CD8^+^ T cells. Venn diagrams illustrate the overlap between DACRs with increased accessibility and upregulated DEGs in *Junb*-deficient CD8^+^ T cells (D), as well as DACRs with decreased accessibility and downregulated DEGs (E). Genome browser images showing downregulated DACRs at the *Bcl2l11*, *Foxo1*, *Foxo3*, *Tcf7*, and *Bcl2l11* loci (F) and upregulated DACRs at the *Pdcd1*, *Havcr2*, and *Ifng* loci (G) in *Junb*-deficient cells. DACRs affected by JunB deficiency are marked with red boxes.

To better understand the molecular mechanism by which JunB controls gene expression in effector CD8^+^ T cell differentiation, we evaluated the impact of JunB deficiency on chromatin accessibility in naive CD8^+^ T cells activated for 96 h using ATAC-seq analysis. This revealed that JunB deficiency led to a decrease in chromatin accessibility in 7079 accessible chromatin regions (ACRs) and an increase in 374 ACRs (log_2_ fold change >0.5, false discovery rate <0.05) (Fig. 7A). Motif enrichment analysis of the DACRs revealed a significant enrichment of AP-1-binding motifs in DACRs that exhibited reduced chromatin accessibility in *Junb*-deficient cells (Fig. 7B). On the other hand, motifs for NR4A1 and RUNX, but not AP-1, were enriched in the DACRs that exhibited increased chromatin accessibility in *Junb*-deficient cells (Fig. 7C). These results imply that JunB directly promotes chromatin accessibility at a subset of loci bound by AP-1, while it indirectly inhibits accessibility at another subset of loci regulated by NR4A1 and RUNX in cytotoxic CD8^+^ T cell responses.

Integrated analysis of our RNA-seq and ATAC-seq data revealed that JunB deficiency reduced chromatin accessibility at 28.2% (120 out of 425) of loci whose expression was upregulated by JunB, including *Foxo1*, *Foxo3*, and *Tcf7* (Fig. 7D and F). In contrast, JunB deficiency increased accessibility at only 0.2% (19 out of 526) of loci whose expression was downregulated by JunB, including *Pdcd1*, *Havcr2*, and *Ifng* (Fig. 7E and G). Notably, the chromatin at the −23.8 kb region of *Pdcd1*, which is known as an enhancer region specifically activated in exhausted T cells (54), exhibited enhanced accessibility in *Junb*-deficient cells (Fig. 7G). Additionally, we found that chromatin accessibility at the −8.3 kb region of *Bcl2l11*, a region reported to be regulated by BATF (55), was decreased by JunB deficiency (Fig. 7F). Comparing with previously reported ChIP-seq data for JunB and BATF in effector CD8^+^ T cells (20), we observed low or background levels of ChIP-seq peaks for JunB and BATF in ACRs affected by JunB deficiency at the *Pdcd1* and *Havcr2* loci (Supplementary Figure 7A and B). These results suggest that JunB contributes to the transcriptional program of cytotoxic CD8^+^ T cell responses, partly through chromatin regulation.

## Discussion

This study demonstrated that JunB is essential for CD8^+^ T cell responses to acute infections. Our data indicate that JunB deficiency hinders clonal expansion of CD8^+^ T cells in response to *L. monocytogenes* infection, resulting in failure to generate both effector and memory CD8^+^ T cells (Fig. 2A–D). Impaired clonal expansion of *Junb*-deficient CD8^+^ T cells is accompanied by increased apoptosis and a marked decrease in the ratio of proliferating effector cells to naive- or memory-like cells in the early phase of responses (Figs 2D and 3B, D). Thus, JunB appears to be required for optimal activation and/or early fate decisions of naive CD8^+^ T cells to differentiate into SLECs. Considering similar defects observed in *Batf*- or *Irf4*-deficient CD8^+^ T cells (20, 29, 30), JunB likely collaborates with BATF and IRF4 to support clonal expansion of effector CD8^+^ T cells. However, in the late phase of responses, unlike BATF and IRF4, which facilitate accumulation of SLECs rather than MPECs (20, 30), JunB deficiency did not affect the ratio between SLECs and MPECs although the cell numbers of both populations were decreased (Fig. 2E). These data suggest that JunB not only directs cell fate decision toward SLEC differentiation but also supports maintenance of differentiated MPECs, thereby contributing to the accumulation of both SLECs and MPECs.

Our *in vitro* findings on the role of JunB in effector CD8^+^ T cell differentiation not only support *in vivo* findings, but also shed light on additional JunB functions. Consistent with *in vivo* observations, JunB inhibits apoptosis and promotes glycolysis *in vitro* (Fig. 4B–E). In contrast, although the proportion of cells expressing *Mki67* proliferation marker gene is decreased in CD8^+^ T cells activated *in vivo* (Fig. 3E), CD8^+^ T cells activated *in vitro* exhibited normal *Mki67* expression and dye dilution (Fig. 4A). The different effects of JunB deficiency on *Mki67* expression *in vitro* and *in vivo* imply that conditions of T cell activation, such as the intensity of stimulatory signals, may influence the requirement for JunB in promoting effector CD8^+^ T cell proliferation. Moreover, although our *in vivo* data suggest that JunB promotes the generation of effector CD8^+^ T cells expressing *Gzmb* mRNA (Fig. 3E), *in vitro* data indicate that like BATF (20), JunB inhibits the expression of *Gzmb* as well as *Ifng* (Fig. 5A and D). Hence, JunB may contribute to the previously suggested role of BATF in acting as a checkpoint to prevent the overproduction of effector molecules during the early differentiation of effector CD8^+^ T cells (20).

This study addresses functional relationships between JunB, BATF, and IRF4 in effector CD8^+^ T cell differentiation, using a comparison of DEGs we found in *Junb*-deficient cells and those reported in *Batf*- or *Irf4*-deficient cells (20, 30). Despite notable differences in experimental settings for *in vitro* effector CD8^+^ T cell differentiation and transcriptome analysis across studies, we found that JunB regulates expression of a substantial number of genes that are controlled by BATF and IRF4 (Fig. 5C). For example, like BATF and/or IRF4, JunB promotes expression of genes related to effector and memory CD8^+^ T cell differentiation (*Il12rb2*, *Runx3*, *Tcf7*, and *Foxo3*) and glycolysis (*Eno2* and *Aldoa*) (Fig. 5D). In contrast, JunB is not involved in regulation of expression of some key genes upregulated by BATF and IRF4, such as *Tbx21* and *Prdm1* in CD8^+^ T cells activated *in vitro* (Fig. 5A). Thus, transcriptional regulatory activity of BATF and IRF4 is supported by JunB in a target-gene-dependent manner in effector CD8^+^ T cell differentiation.

Our data suggest that JunB directly promotes chromatin accessibility at various loci. Notably, as observed at loci such as *Foxo1* and *Tcf7*, about 30% of genes upregulated by JunB were associated with JunB-mediated enhanced chromatin accessibility (Fig. 7D and F). Additionally, as observed at the *Bcl2l11* locus, the JunB-mediated increase of chromatin accessibility is also associated with the downregulation of gene expression (Fig. 7F). BATF facilitates chromatin remodeling in the differentiation of effector CD8^+^ T cells (31) and Th17 cells (32). Studies of Th17 differentiation suggest that BATF can bind to closed chromatin to increase its accessibility, thereby promoting the binding of other TFs to specific loci to facilitate their expression in T cells (56). Comparing chromatin regions regulated by JunB and BATF in effector CD8^+^ T cell differentiation is currently challenging because markedly different experimental settings have been used in each analysis. Nevertheless, we observed that JunB controls accessibility in a subset of chromatin regions, such as the *Bcl2l11* region, regulated by BATF *in vivo* during the CD8^+^ T cell response to acute infection. Future studies should extend this comparative analysis in a common experimental setting to further address whether JunB is required for BATF-mediated regulation of chromatin remodeling in effector CD8^+^ T cell differentiation.

Interestingly, JunB also appears to indirectly inhibit chromatin accessibility at some loci, including those with binding sites for NR4A1 and RUNX (Fig. 7C). The contribution of this chromatin regulation to transcriptional regulation remains largely unclear. However, we discovered that JunB inhibits chromatin accessibility in the −23.8 kb region of *Pdcd1* and −0.3 kb region of *Havcr2*, along with expression of PD-1 and Tim3 (Figs 6A and 7G). The −23.8 kb region of *Pdcd1* is the enhancer specifically activated in exhausted T cells (54), serving as an irreversible epigenetic mark that leads to sustained PD-1 expression (54, 57). Unlike JunB, BATF does not appear to be involved in the regulation of the −23.8 kb region of *Pdcd1* (31), suggesting that JunB may serve this function together with AP-1 factors other than BATF, although the underlying mechanism remains unknown. Given that PD-1 induced in early CD8^+^ T cell response to acute infection inhibits cytotoxic CD8^+^ T cell differentiation (53), increased PD-1 expression caused by JunB deficiency may also be involved in impaired CD8^+^ T cell responses. The mechanism by which JunB indirectly inhibits chromatin accessibility is unknown. An interesting question for future studies is whether JunB directly or indirectly modulates the expression or activity of NR4A1 and c-Jun, which promote and inhibit T cell exhaustion, respectively (58, 59).

We observed that JunB expression is induced by TCR and CD28 co-stimulatory signaling in activation of naive CD8^+^ T cells, consistent with previous observations in CD4^+^ T cells (36, 37). We also found that, like IRF4 (29, 30), JunB expression is positively associated with TCR signal strength (Fig. 1C). Interestingly, JunB appears to be required for full induction of IRF4 in TCR-stimulated CD8^+^ T cells (Supplementary Figure 2C and D). This contrasts with observations that JunB is dispensable for IRF4 expression in CD4^+^ T helper and effector Treg cells (33, 34, 37). The cell-type-specific requirement for JunB to enhance IRF4 expression in T cell subsets may be due to the differential availability of other AP-1 TFs that can compensate for the loss of JunB. Our previous studies demonstrated that JunB is essential for IRF4 to bind to a subset of its target genes in CD4^+^ T cells (33, 37). Although whether this is also the case in CD8^+^ T cells remains to be determined, our data showing shared transcriptional regulatory targets for JunB and IRF4 in CD8^+^ T cells (Fig. 5C) support this possibility. As IRF4 promotes the transcriptional program for T cell activation, differentiation, and metabolic reprogramming in a dose-dependent manner (30), modulation of JunB expression in conjunction with IRF4 may constitute the molecular basis for the relationship between TCR signaling strength and the corresponding CD8^+^ T cell response. Furthermore, given that co-stimulation signaling is required for chromatin remodeling at many loci bound by AP-1, as shown in human CD4^+^ T cells (60), our findings suggest that JunB may be involved in chromatin remodeling facilitated by a co-stimulatory signal in CD8^+^ T cells.

This study has several limitations that should be addressed in future studies. First, since all analyses were performed only on samples collected at one or several selected time points, data must be more comprehensive to understand JunB functions in CD8^+^ T cell responses. Second, since our *in vivo* analysis focused on only a single acute infection model with *L. monocytogenes*, further analysis using other infection models, such as lymphocytic choriomeningitis virus and influenza virus, is needed to generalize the conclusions of this study. Third, differences in sampling schemes and experimental settings of RNA-seq and ATAC-seq between this study and other studies made it challenging to accurately identify common or unique target genes for JunB, BATF, and IRF4. Fourth, although we addressed JunB functions in chromatin regulation using analysis of CD8^+^ T cells activated *in vitro*, whether JunB plays similar roles *in vivo* remains to be determined. Although it is difficult to collect a sufficient number of *Junb*-deficient CD8^+^ T cells activated *in vivo* for bulk ATAC-seq analysis, future single-cell ATAC-seq analysis could address this issue.

In summary, we have demonstrated that JunB-dependent transcriptional regulation is important for CD8^+^ T cell responses to acute infection. JunB is transiently induced in antigen-stimulated naive CD8^+^ T cells and regulates expression of genes related to apoptosis, glycolysis, and co-inhibitory receptors, thereby supporting clonal expansion of cytotoxic CD8^+^ T cells. These findings advance our understanding of the transcriptional regulatory mechanism critical for early cytotoxic CD8^+^ T cell responses.

## Supplementary data

Supplementary data are available at *International Immunology* Online.

dxae063_suppl_Supplementary_Figures
