# Supplementary material for: JunB is required for CD8+ T cell responses to acute infections
Source: Int Immunol. 2024 Oct 19;37(4):203–20. doi: 10.1093/intimm/dxae063 (PMC11884676; doi:10.1093/intimm/dxae063)
Supplement: dxae063_suppl_Supplementary_Figures [file dxae063_suppl_supplementary_figures.pdf]

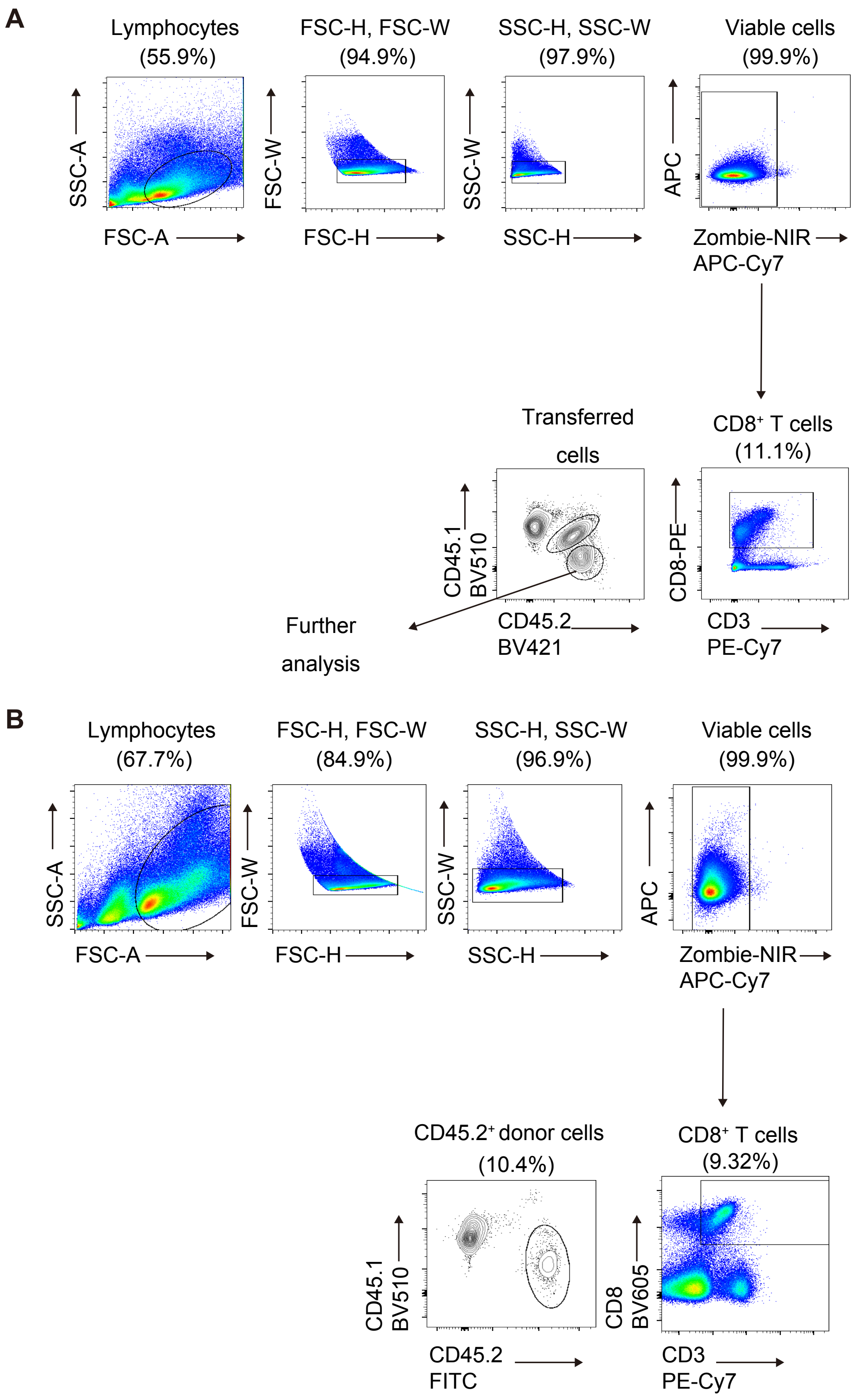

Supplementary Fig.1. (A) Gating strategy for flow cytometry analysis of *Junb*-deficient or control OT-I T cells ($CD45.1^{-}$ $CD45.2^{+}$) co-transferred with WT OT-I T cells ($CD45.1^{+}CD45.2^{+}$) isolated from mice infected with LM-OVA at day 7 post-infection. **(B)** Gating strategy for flow cytometry analysis of transferred control OT-I T cells ($CD45.1^{-}$ $CD45.2^{+}$) from mice infected with LM-OVA at day 5 post-infection.

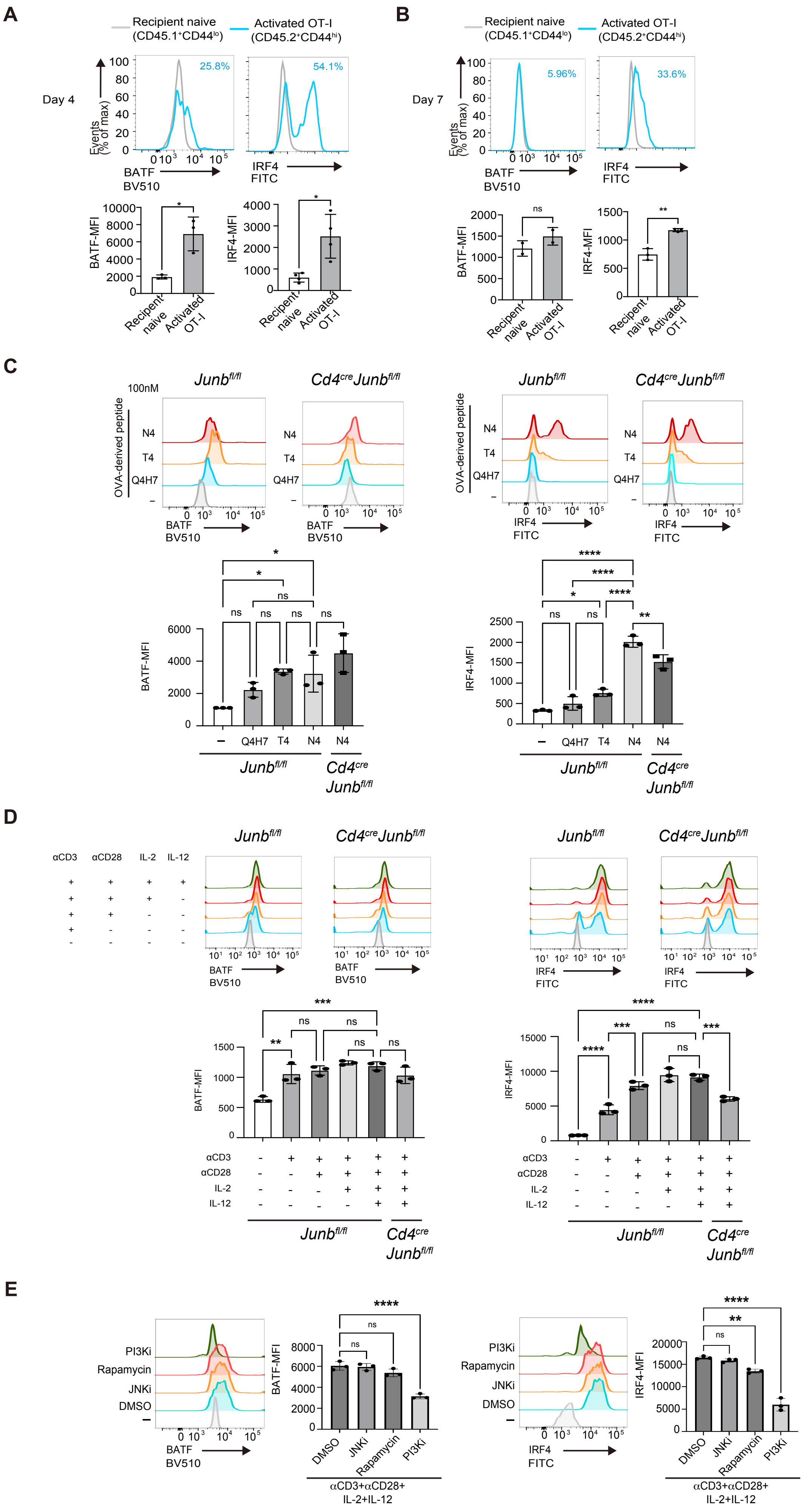

A
Recipient naive (CD45.1⁺CD44lo)
Activated OT-I (CD45.2⁺CD44hi)
Day 4
25.8%
54.1%
Events (% of max)
BATF BV510
IRF4 FITC
BATF-MFI
IRF4-MFI
Recipient naive
Activated OT-I
B
Day 7
5.96%
33.6%
ns
C
Junbfl/fl
Cd4creJunbfl/fl
100nM
OVA-derived peptide
N4
T4
Q4H7
BATF-MFI
IRF4-MFI
D
αCD3
αCD28
IL-2
IL-12
E
PI3Ki
Rapamycin
JNKi
DMSO
DMSO
JNKi
Rapamycin
PI3Ki
αCD3+αCD28+ IL-2+IL-12

Supplementary Fig.2. OT-I T cells ($CD45.2^+$) were transferred into congenic recipient mice ($CD45.1^+$), followed by LM-OVA infection. Cells were isolated from the spleen on Days 4 **(A)** and 7 **(B)** p.i. and were subjected to flow cytometry analysis. Upper panel: representative histograms showing expression of JunB in activated OT-I T cells ($CD45.2^+CD44^{hi}$) or recipient naïve $CD8^+$ T cells ($CD45.1^+CD44^{lo}$). Lower panel: Graph showing percentages of cells expressing IRF4 or BATF in $CD44^{hi}$ OT-I cells and recipient $CD44^{lo}CD8^+$ T cells. Error bars indicate one standard deviation (s.d) (n=3). **p<0.01, ***p<0.001, ****p<0.0001, ns:not significant (unpaired two-tailed student's t-test). **(C)** Control (*Junb*$^{fl/fl}$) or *Junb*-deficient (*Junb*$^{fl/fl}$*Cd4*^{cre}) OT-I T cells were stimulated with irradiated splenocytes pulsed with an OVA-derived peptide (N4) or its variants (T4 or Q4H7). **(D)** Control (*Junb*$^{fl/fl}$) or *Junb*-deficient (*Junb*$^{fl/fl}$*Cd4*^{cre}) naïve $CD8^+$ T cells were activated by anti-CD3 antibody with or without anti-CD28 antibody in the presence or absence of cytokines IL-2 and IL-12. **(E)** Naive $CD8^+$ T cells were activated by anti-CD3 antibody with or without anti-CD28 antibody in the presence of pharmacological inhibitors for JNK (JNKi), PI3K (PI3Ki), or mTOR (rapamycin). **(C-E)** At 24 h post activation, expression of BATF and IRF4 was analyzed by flow cytometry. In each panel, flow cytometry histograms show BATF or IRF4 expression, and bar graphs show MFI of BATF and IRF4 expression. Error bars indicate s.d (n=3). **p<0.01, ***p<0.001, ****p<0.0001, ns: not significant (one-way ANOVA with Bonferroni's multiple comparison tests). Data are representative of two independent experiments.

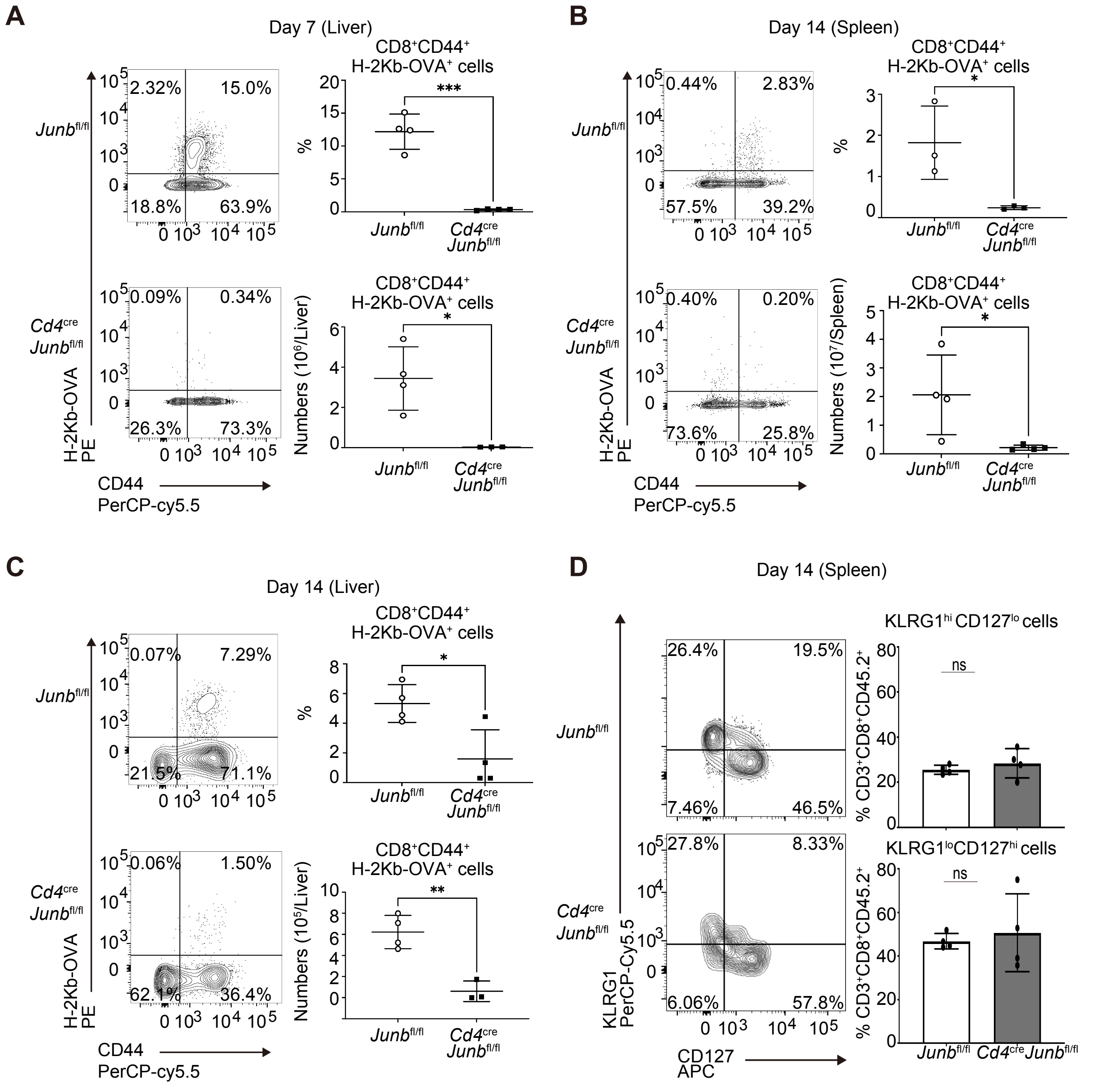

Supplementary Fig.3. Control (*Junb*$^{fl/fl}$) or *Junb*-deficient (*Junb*$^{fl/fl}$*Cd4*^{cre}) mice were infected with LM-OVA ($5x10^3$ CFU). Cells were isolated from livers on Day 7 **(A)** and from spleens **(B)** or livers **(C)** on Day 14, then subjected to flow cytometry analysis of binding to H-2Kb-restricted OVA tetramers. Left: Represent plots showing binding of OVA tetramers (H-2Kb-OVA) and expression of CD44 in cells gated on $CD3^+CD8^+$. Right: graphs showing proportions of H-2Kb-OVA^+CD44^+ cells in $CD8^+$ T cells (top) and their absolute numbers (bottom). **(D)** Flow cytometry analysis of expression of KLRG1 and CD127 in OT-I T cells isolated from spleens on Day 14. Left: representative plots. Right: graphs showing percentages of SLECs ($KLRG1^{hi}CD127^{lo}$) and MPECs ($KLRG1^{lo}CD127^{hi}$). Error bars indicate one s.d (n=4 per group). *p<0.05, **p<0.01, ***p<0.001(unpaired two-tailed Student's t-test). Data represent two independent experiments.

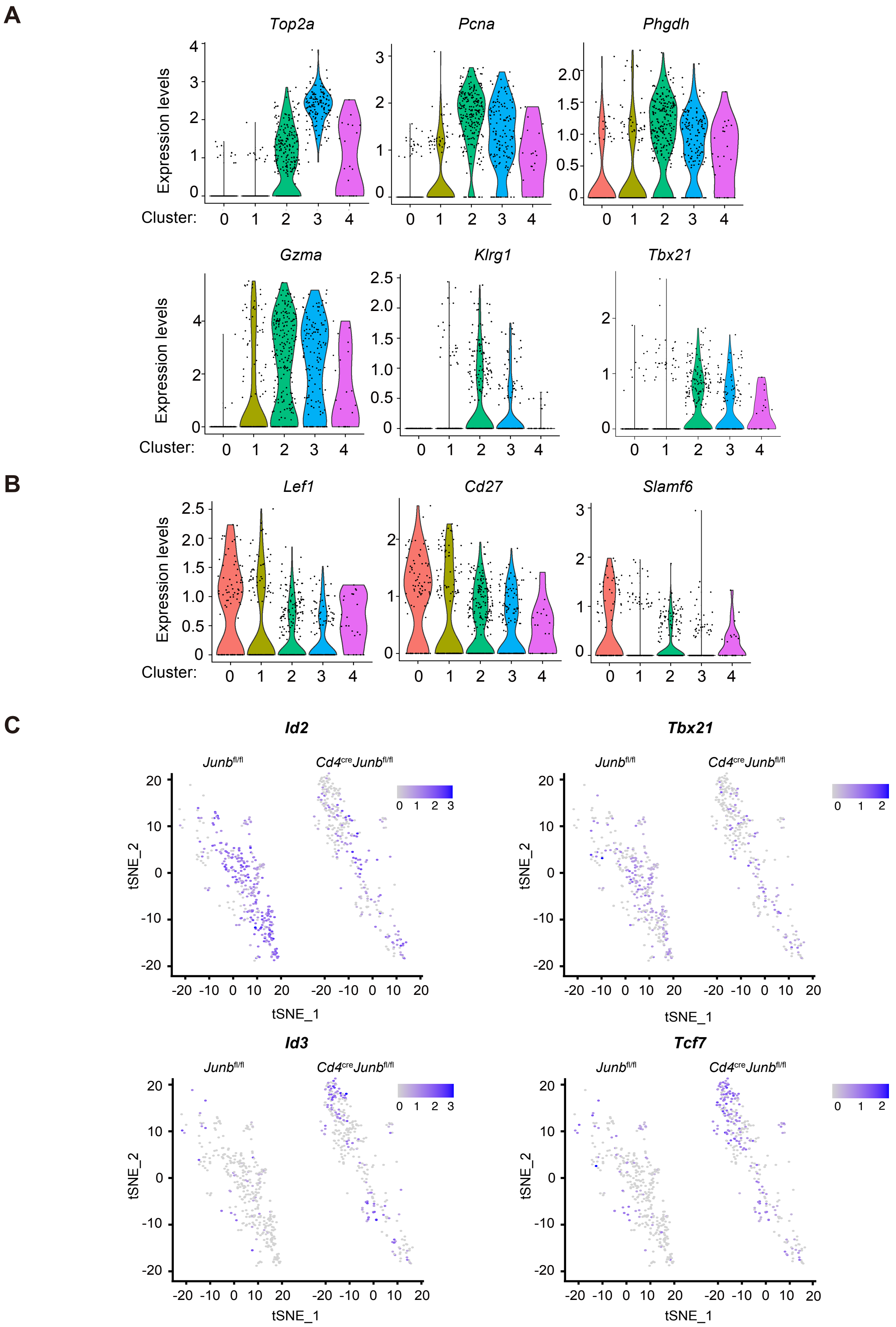

Supplementary Fig.4. Control (*Junb*$^{fl/fl}$) or *Junb*-deficient (*Junb*$^{fl/fl}$*Cd4*^{cre}) OT-I cells (CD45.2^{+}) were transferred into congenic recipient mice (CD45.2^{+}), followed by infection with LM-OVA. On Day 5, living OT-I T cells (CD45.2^{+}) were sorted from splenocytes and subjected to scRNA-seq analysis. t-SNE clustering analysis was performed. **(A, B)** Violin plots show the expression of representative genes highly expressed in clusters 2 and 3 **(A)** and those highly expressed in clusters 0 and/or 1 **(B)**. Each gene name is shown in the plot. **(C)** Expression of *Id2*, *Id3*, *Tbx21*, and *Tcf7* in control or *Junb*-deficient cells was visualized on t-SNE plots. Colour scales indicate expression levels.

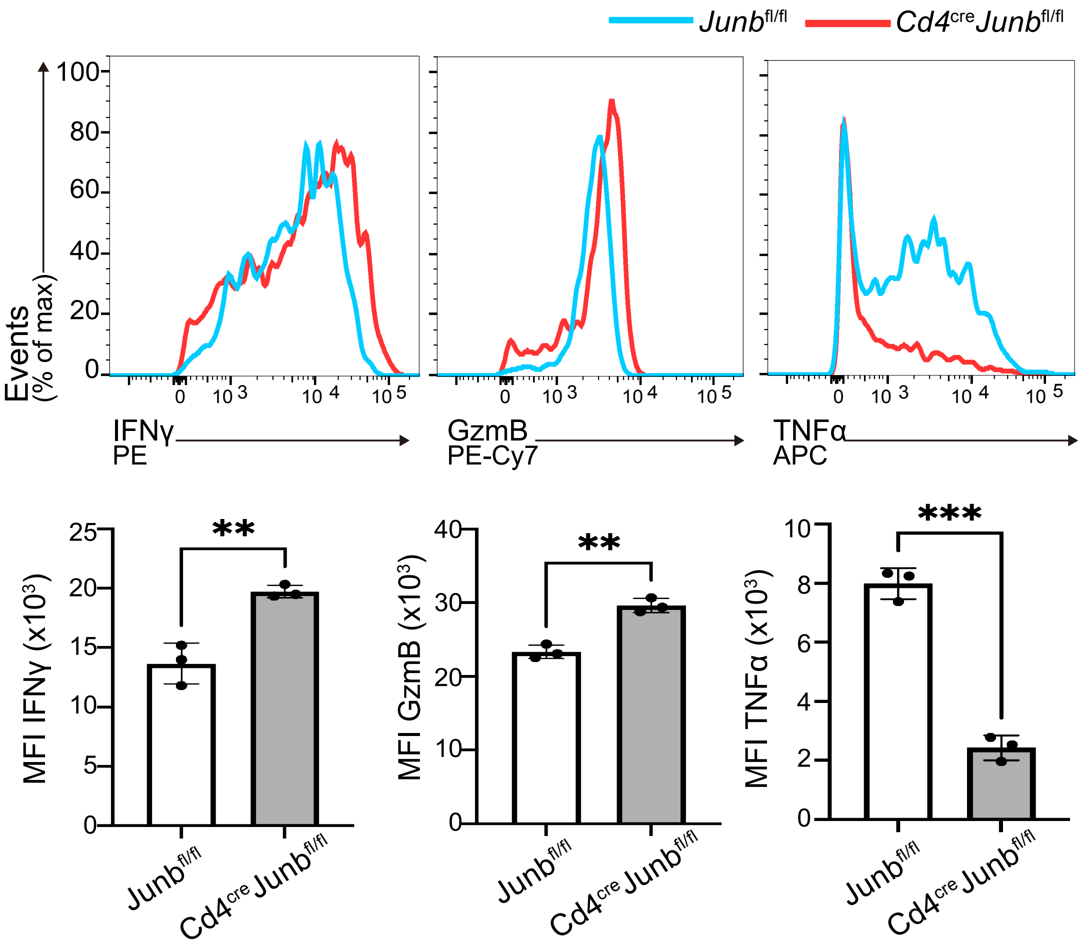

Supplementary Fig.5. Control (*Junb*$^{fl/fl}$) or *Junb*-deficient (*Junb*$^{fl/fl}$*Cd4*^{cre}) naive CD8^{+} T cells were activated with anti-CD3, anti-CD28, IL-2, and IL-12 *in vitro*. Expression of IFN-γ , GzmB, TNF-α at 96 h after activation was analyzed by flow cytometry. Top: representative histograms. Bottom: graphs showing MFI of IFN-γ, GzmB, and TNF-α expression. Error bars indicate s.d (n=3). **p<0.01, ***p<0.001 (unpaired two-tailed student's t-test). Data are representative of two independent experiments.

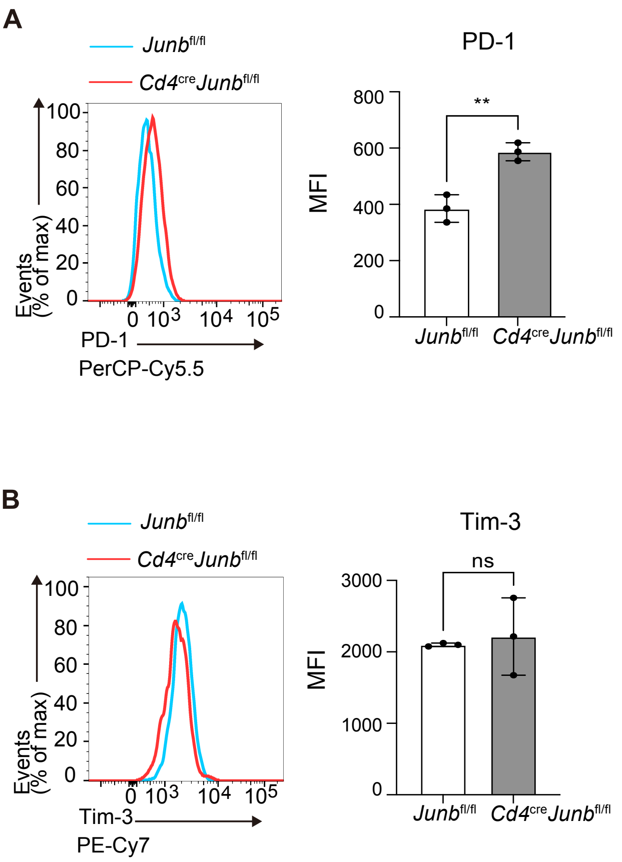

Supplementary Fig.6. Control (*Junb*$^{fl/fl}$) or *Junb*-deficient (*Junb*$^{fl/fl}$*Cd4*^{Cre}) OT-I T cells (CD45.2^{+}) were transferred into congenic recipient mice (CD45.1^{+}), followed by infection with LM-OVA. On day 5, splenocytes were collected and subjected to flow cytometry analysis. Expression of PD-1 **(A)** and Tim-3 **(B)** in OT-I T cells gated on CD45.2^{+} was analyzed. Left: representative histograms. Right: Graphs show MFI. Error bars indicate s.d. (n=3 per group). **$p < 0.01$, ***$p < 0.001$, ****$p < 0.0001$ (unpaired two-tailed Student's t-test).

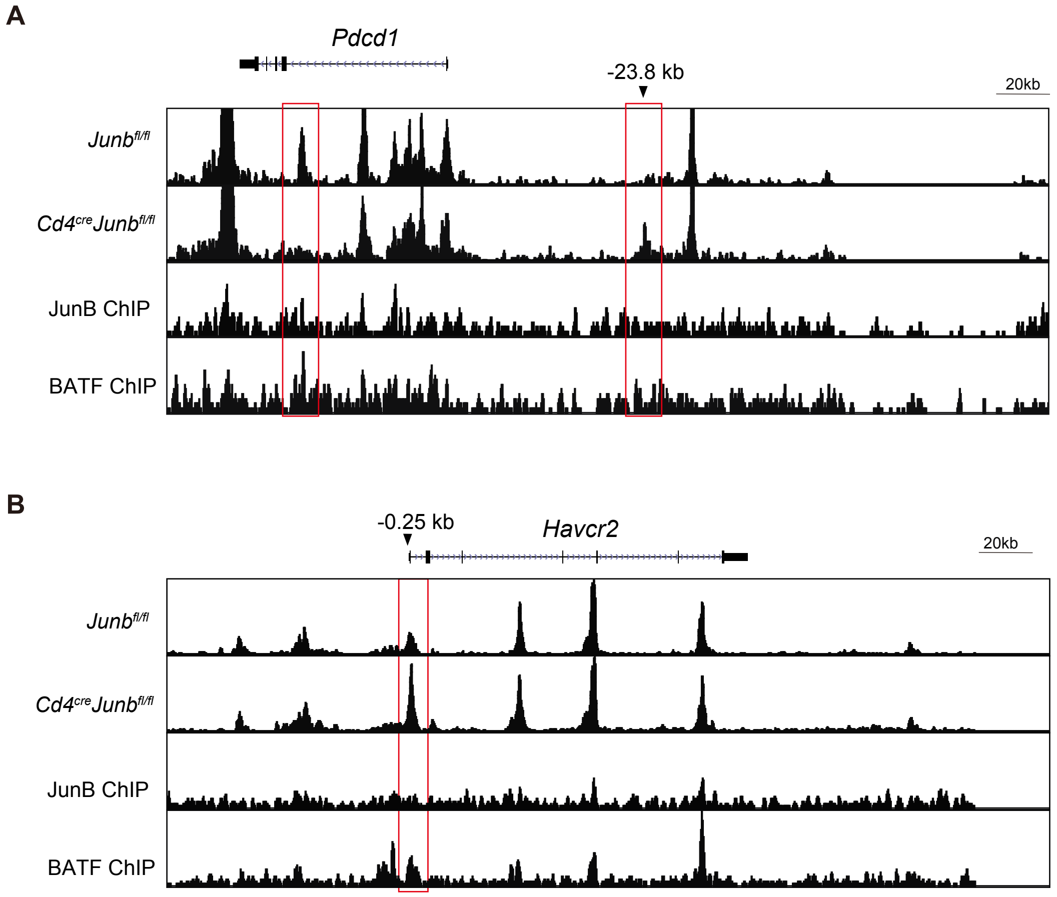

Supplementary Fig.7. Control (*Junb*$^{fl/fl}$) or *Junb*-deficient (*Junb*$^{fl/fl}$*Cd4*^{Cre}) naïve CD8^{+} T cells were activated by anti-CD3 and anti-CD28 antibodies, IL2, and IL-12 *in vitro* for 96 h and subjected to ATAC-seq analysis. Genome browser images showing DACRs and genome-binding regions of JunB and BATF at *Pdcd1* **(A)** and *Havcr2* **(B)** loci of control and *Junb*-deficient cells. DACRs affected by JunB deficiency are marked with red boxes. ChIP-seq data were obtained from a previous study, GSE54191. ATAC-seq data are available at PRJDB17429.
